# Probing morphological, genetic and metabolomic changes of in vitro embryo development in a microfluidic device

**DOI:** 10.1002/btpr.3194

**Published:** 2021-07-29

**Authors:** Vanessa Mancini, Paul J. McKeegan, Alexandra C. Schrimpe‐Rutledge, Simona G. Codreanu, Stacy D. Sherrod, John A. McLean, Helen M. Picton, Virginia Pensabene

**Affiliations:** ^1^ School of Electronic and Electrical Engineering University of Leeds Leeds UK; ^2^ Reproduction and Early Development Research Group, Discovery and Translational Science Department, Leeds Institute of Cardiovascular and Metabolic Medicine, School of Medicine University of Leeds UK; ^3^ Centre for Anatomical and Human Sciences, Hull York Medical School University of Hull Hull UK; ^4^ Center for Innovative Technology (CIT), Department of Chemistry Vanderbilt University Nashville Tennessee USA; ^5^ Leeds Institute of Medical Research University of Leeds UK

**Keywords:** assisted reproductive technologies, embryo culture, IVF, mice, microfluidics

## Abstract

Assisted reproduction technologies for clinical and research purposes rely on a brief in vitro embryo culture which, despite decades of progress, remain suboptimal in comparison to the physiological environment. One promising tool to improve this technique is the development of bespoke microfluidic chambers. Here we present and validate a new microfluidic device in polydimethylsiloxane (PDMS) for the culture of early mouse embryos. Device material and design resulted embryo compatible and elicit minimal stress. Blastocyst formation, hatching, attachment and outgrowth formation on fibronectin‐coated devices were similar to traditional microdrop methods. Total blastocyst cell number and allocation to the trophectoderm and inner cell mass lineages were unaffected. The devices were designed for culture of 10–12 embryos. Development rates, mitochondrial polarization and metabolic turnover of key energy substrates glucose, pyruvate and lactate were consistent with groups of 10 embryos in microdrop controls. Increasing group size to 40 embryos per device was associated with increased variation in development rates and altered metabolism. Device culture did not perturb blastocyst gene expression but did elicit changes in embryo metabolome, which can be ascribed to substrate leaching from PDMS and warrant further investigation.

## 1 INTRODUCTION

This work has impact on microfluidics, embryology and metabolomics techniques. With the final goal to develop an alternative, simplified embryo culture system to improve the efficiency of in vitro fertilization (IVF) procedures for breeding genetically altered mice, we completed a thorough study of preimplantation embryo development applying an exhaustive set of analytic techniques. We observed subtle, different developmental characteristics of embryos cultured in drops under oil or in a microfluidic, closed conduit. These data are essential for planning carefully the embryo transfer trial, in compliance with 3Rs principle, to confirm the impact of the microfluidic system on the embryo quality and to extend its application to other research/domestic species and humans. We linked morphokinetics, genetic and metabolomic profiles with chemical and physical characteristics of the manufacturing plastics. These data are informative for the research community in microfluidics and embryology, providing additional understanding on the safety of manufacturing plastics in IVF.

Genetically altered (GA) mice are widely used as models for developing clinical practice and furthering our understanding of human and animal reproduction and diseases. Almost 50% of the total amount of animals used for scientific research are genetically modified animals, the majority of which are mice.^1^ In recent years a resurgence in the use of GA mouse models in medical research and pharmaceutical industry has occurred thanks to the introduction of sequencing methods, and our ability to combine genetic engineering technologies^2^ such as CRISPR/Cas9, with methods used for microinjection, nuclear transfer as well as IVF, embryo and stem cell culture and derivation. The animal facilities for breeding GA mice are increasingly reliant on assisted reproduction technologies (ART) to generate embryos in vivo and in vitro for manipulation and cryopreservation to build research banks of GA embryos or to distribute GA embryos or animals to the research community.^3^, ^4^ High standards of animal welfare are required to support the processes involved in the generation of GA animals for research in order to generate, robust, high‐quality data.^5^ Many mouse GA facilities therefore need to implement significant changes in their breeding protocols and production methods in order to maximize efficiency, reduce animal suffering, improve throughput and increase pregnancy and birth rates of genetically modified animals.^6^, ^7^, ^8^

**FIGURE 1:**
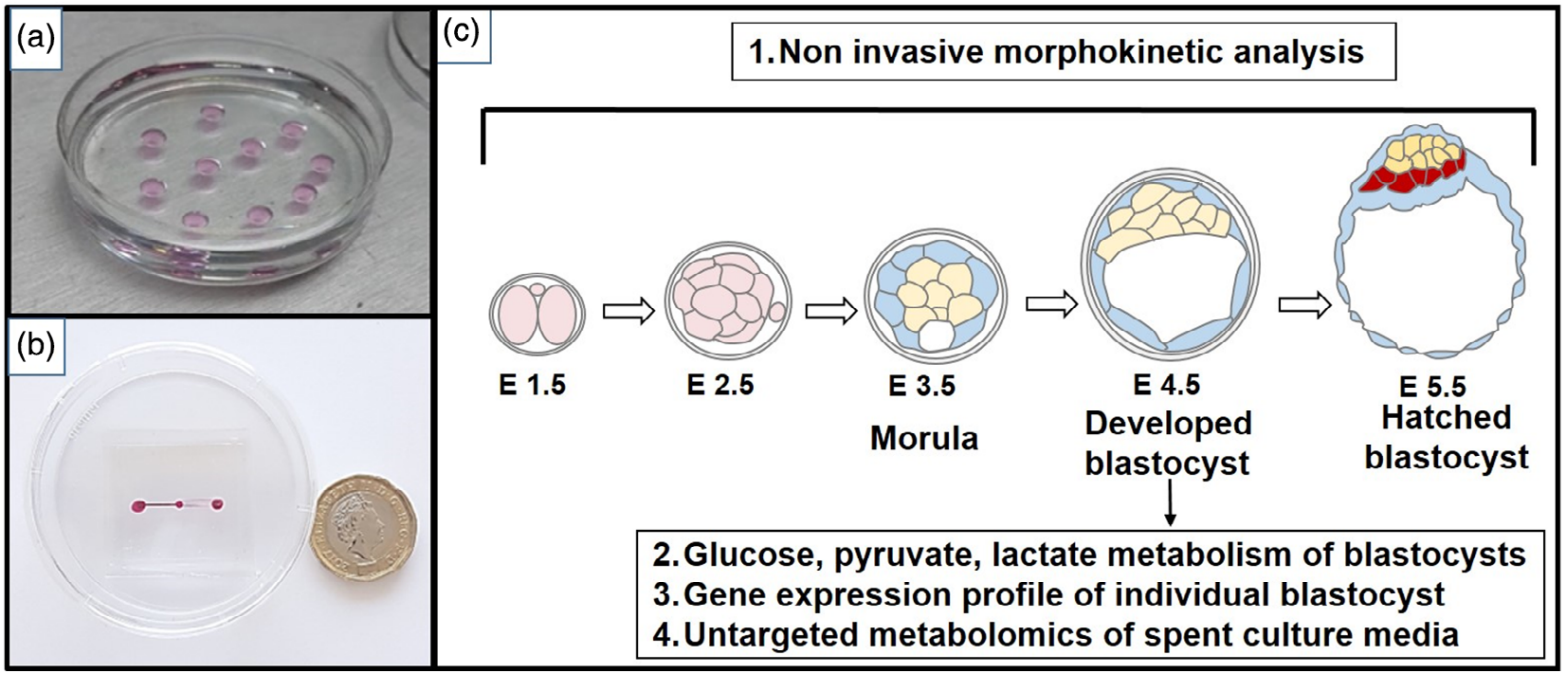
(a) Traditional microdrop culture in a 60 mm dish. (b) Image of the fabricated PDMS microfluidic device sitting within a standard 60 mm culture dish. Red dye indicates inlet and outlet ports and microfluidic channels. Image shown next to a 1 GB pound coin for scale. (c) Schematic of in vitro murine embryo development and noninvasive analytical methods used to monitor and stage the embryos during culture and to evaluate single embryo quality

The in vitro production of mouse embryos typically involves the culture of multiple embryos in 5–100 μl microdrops of specialized embryo culture medium^9^ in Petri dishes covered with a layer of mineral oil to prevent media evaporation and associated changes in pH and osmolarity (Figure 1a). Single cell, fertilized zygotes or early cleavage staged preimplantation embryos derived in vivo or in vitro are transferred to sterile microdrops of pre‐equilibrated, defined culture media and allowed to develop undisturbed in a humidified and gassed incubator environment for 4–5 days until they reach the blastocyst stage of development.^10^ When ready for transfer, mouse blastocysts are aspirated and injected in the oviduct or uterus by traditional surgical embryo transfer (SET) techniques or by nonsurgical embryo transfer (NSET).^7^ Embryo handling for both SET and NSET transfers involves manual pipetting, which is labor intensive, time consuming, and complicated by the presence of the mineral oil over layer in the culture dish. First implemented in 2009, NSET techniques are now adopted globally and are of similar or improved efficiency to SET.^6^, ^7^, ^11^ Considerable research effort in a range of species has confirmed that embryo development and implantation efficiency are highly variable and are critically dependent on a range of factors that include the embryo development stage at transfer^12^ and the creation and use of species specific, optimal culture environments.^13^ Higher implantation and pregnancy rates post transfer have been reported when culturing embryos to the morula or blastocyst stage in vitro prior to transfer, a procedure requiring up to 4 days of culture in the mouse and 5–8 days in other mammalian species.^14^, ^15^, ^16^

Since the 1990s, microfluidics has been proposed as a new approach to optimize the specialized in vitro requirements for successful ART. In 2013, Swain summarized the advantages and limitations of the microfluidic approach for clinical procedures in ART (these include oocyte maturation, manipulation, embryo culture, cryopreservation and noninvasive quality assessment).^17^ LeGac also reported an overview of existing microfluidic platforms specifically used for embryo culture and characterization, discussing both intrinsic benefits and factors which currently limit the adoption of those innovative techniques in IVF clinical laboratories.^18^ Moreover, Esteves et al. assessed the beneficial effect of volume reduction on single and group embryo culture using a microfluidic chamber that supported improved blastocyst development without altering birth rate.^19^ Later in 2017, Ferraz et al. attempted to create an oviduct‐on‐a‐chip platform using 3D printing technology.^20^ Although this system was successful for sperm penetration of bovine oocytes and reduction of abnormal fertilization compared to standard IVF systems, the material used for 3D printing resulted toxic to fertilized oocytes.^21^ The same group recently developed a polydimethylsiloxane (PDMS) oviduct‐on‐a‐chip device for the culture of oviductal epithelial cells and production of bovine zygotes.^22^ However, none of these systems has been translated in commercially available devices. While the interest in the use of these microfluidic systems is still high, extensive scientific analyses to assess safety, consistency and accuracy remain and the long‐term impact of culturing embryos in microfluidic devices is not completely understood.

In recent years, the variable developmental competence (quality) of embryos produced using ARTs has led to the development of a range of invasive and noninvasive clinical and research methods that can be used to directly quantify and hence predict embryo implantation and pregnancy potential.^23^ These embryo quality assays can now be used to generate an in‐depth understanding of the impact of microfluidic culture and the reduced culture volumes on the health and developmental potential of in vitro‐derived mouse embryos. Morphokinetics (embryo morphology, cleavage rate, timing, cell number and fragmentation),^23^, ^24^, ^25^, ^26^, ^27^ embryo energy metabolism,^28^, ^29^, ^30^ blastocyst hatching and outgrowth rates,^31^ blastocyst production and cell count, and blastocyst cell allocation to the inner cell mass versus the trophectoderm^32^, ^33^, ^34^ as well as in‐depth molecular analysis of trophectoderm biopsies represent biomarkers of blastocyst competence that can be evaluated in vitro and can be used to optimize the microfluidic technology before moving to in vivo testing, thus avoiding unnecessary sacrifice of animals.

While some of these methods have been used for validation of microfluidic prototypes, metabolic, genetic and epigenetic signatures of embryos developed in microfluidic devices have never been compared with those of embryos cultured in traditional microdrops. Preimplantation embryo development is defined by specific patterns in gene expression associated with cell divisions, starting from the fertilized zygote and progressing through embryonic genome activation to the blastocyst stage of development. The sequence takes approximately 4.5 days in mice. In the last decade, expression patterns of thousands of genes with specific functions during mouse embryo preimplantation development have been identified.^35^, ^36^ Analysis of candidate genes known to be involved in trophoblast differentiation (such as caudal type homeobox *Cdx‐2*,^37^ TEA domain family member 4 *Tead4* ^38^ and E74‐like factor 5 *Elf5* ^39^) and inner cell mass (ICM)/epiblast development (such as octamer‐binding transcription factor *Oct‐4* ^40^ and SRY [sex determining region Y]‐box 2 *Sox2*), or transcriptional repressors such as the methylcytosine binding protein genes Mbd,^41^ can be used to provide valuable insights into the potential effects of the microfluidic environment on key markers of embryo development and health.^42^, ^43^

In the current study we developed and fabricated a novel, oil‐free disposable microfluidic device in PDMS, in which fertilized 1 cell/two pronuclei stage murine zygotes can be grown to the expanded blastocyst stage in vitro and retrieved for subsequent embryo transfer (Figure 1b). The requirement for manual pipetting of embryos is reduced compared to transfer between microdrops under oil, due to the relative ease of loading of embryos in a simple step, as a group, or individually, into the device chamber by flow of media from the inlet to outlet. Further washes of embryos or media changes, if required, can be performed by replacing the media and without direct movement of the embryos. In order to minimize any fluid dynamic shear stress during embryo handling and their injection into the device, the microfluidic design was optimized using finite element modeling; the model also verified the efficiency of the embryo loading and nutrient diffusion in the system. The potential toxicity of PDMS was assessed by performing the mouse embryo assay, in which embryo cleavage, blastocyst development, hatching and outgrowth rate were used as predictive indexes of embryo health and implantation potential. The embryo culture conditions were optimized by studying the effect of group embryo culture on blastocyst development rate, performing metabolic profiling and determining mitochondrial polarization ratios. Furthermore, the gene expression profiles of blastocysts developed inside the system were compared to those cultured in traditional microdrops using real‐time PCR analysis (Figure 1c). These data were used to exclude potential genetic alterations induced by the different environment and culture methods. Finally, global untargeted metabolomics was used to identify PDMS‐released compounds from culture media extracted from the microfluidic device at different time points (24 h and 5 days).

## 2 MATERIALS AND METHODS

### 2.1 Microfluidic device flow and shear stress analysis

All chemicals were purchased from Sigma Aldrich (St Louis, MO, USA) unless specified otherwise. COMSOL Multiphysics 5.2a was used to evaluate and compare flow rate, velocity field and predict shear stress as function of microfluidic device geometry. To generate a 3D model, the microfluidic design was first created by using computer‐aided design software (Autodesk AutoCAD 2017). The design geometry was then imported into a COMSOL library. The fluid inside the device was simulated as an incompressible, homogeneous, Newtonian fluid with density (ρ = 1000 kg m^−3^) and viscosity (μ = 1 × 10^−3^ Pa s).^44^ Flow was created by manually loading a solution of 4.8 μm fluorescent polystyrene beads using a Flexipette with 170 μm tip (EZ‐Grip, RI, Cooper Surgical, Denmark). The maximal velocity in the inlet channel was estimated to be respectively about 0.4 mm/s (as average of 10 measurements) which corresponds to a flow rate equal to 1.17 μl/min (Re: 0.087). The devices showed a widely lower flow rate compared to the theoretically derived harmful value previously calculated (13.91 μl/min).The estimated inlet velocity was applied to a COMSOL model to predict the shear stress in the device during embryo loading (Figure S1). Critical values of shear stress (~3.5 dyn cm^−2^) were only found on the edges at the interface between culture chamber and outlet channels, areas that embryos cannot reach because of the narrow outlet channels section.

Once velocity has been assessed, to prove the computational model results and to better characterize flow profile inside the microfluidic chambers a solution of fluorescein (0.05 mg/ml) was flowed into the chamber previously filled with water.

### 2.2 Microfluidic device fabrication and preparation for culture

Microfluidic devices were fabricated in polydimethylsiloxane (PDMS) using a 10:1 PDMS prepolymer: curing agent mixture (Sylgard® 184, Dow Corning, MI, USA), and completing curing for minimum 4 h. This microfluidic structure was obtained by bonding together two layers of PDMS (Figure 1b), where microchambers and microchannels are defined by standard soft lithography techniques. Once assembled, the devices were immediately filled with sterile tissue culture water and stored for up to 4 days closed at 4°C to preserve hydrophilicity. Before embryo culture, devices were sterilized by exposure to UV light (254 nm wavelength for 30 min). Devices were then prepared by drawing 10 μl KSOMaa (Millipore, UK) from the inlet through the channel and chamber. Devices were then primed by overnight incubation with 10 μl KSOMaa media drops added to the inlet and outlet ports in a benchtop MINC™ Mini incubator (Cook, Aus) at 37°C in humidified 5% CO_2_, 5% O_2_, 90% N_2_. The microfluidic devices were placed inside 60 mm culture dishes and surrounded with embryo tested sterile water to prevent media evaporation. Under these conditions, no significant evaporation was detected after the culture period. To load the device, embryos were placed in the inlet chamber and media (approx. 2 μl) was drawn through from the channel outlet using an embryo handling Flexipette with 170 μm tip until all embryos entered the central chamber (Video S1 loading and Video S2 retrieval). Ten microliter drops of pre‐equilibrated potassium‐supplemented simplex optimization media (KSOM) were then added to channel inlet and outlet before culture at 37°C under 5% CO_2_, 5% O_2_ in humidified nitrogen. The device culture chamber was 400 nl, with the ratio of embryo: medium within the chamber 1:0.04 rather than the standard 1:1 of the microdrop controls.

A total of 46 devices were used for embryo culture in experiments 1 and 2 and additional 30 devices for RT‐PCR and metabolomic analysis in experiments 3 and 4.

### 2.3 Embryo thawing and in vitro embryo culture in microdrops

Cryopreserved embryos (strain C57BL/6N) for Experiments 1 and 2 were supplied at zygote and 2‐cell stages by MRC Harwell, UK. 1‐cell murine embryos (B6C3F1xB6D2F1 strain, EmbryoTech, USA) were used for experiments 3 and 4. On the first day of culture, murine presumptive zygotes were thawed following established protocols. Briefly, embryo straws were held in air for 30 s, then plunged into room temperature water until the contents had visibly thawed (around 10 s). The straws were cut at the seal and the plug bisected before pushing the contents into a 60 mm IVF hydrophobic culture dish. Embryos were incubated for 5 min before 2, 5 min washes in 100 μl M2 medium at 37°C. Embryos were then washed through 3, 500 μl wells of pre‐equilibrated KSOMaa before transfer to devices or culture microdrops. Culture microdrops were 1 μl/embryo in 35 mm hydrophobic IVF certified dishes (Nunc), covered with 5 ml of BioUltra mineral oil from Sigma Aldrich.

### 2.4 Experiment 1: Cell count, outgrowth assay

Embryos were cultured in microdrops and microfluidic devices in groups of 10. Sixty embryos were cultured in each of five replicate cultures for a total of 300 embryos. Cell counts at the end of culture were performed using the method described by Thouas et al.^45^ Briefly zona‐intact blastocysts were first incubated in 500 μl of Dulbeccos PBS with 1% Triton X‐100 and 100 μg/ml propidium iodide for 5 s resulting in a labeled trophectoderm with red fluorescent signal. Blastocysts were then immediately transferred into 500 μl of fixative solution (100% ethanol with 25 μg/ml Hoechst 33258) and stored at 4°C overnight. Fixed and stained blastocysts were then mounted onto a glass microscope slide and gently flattened with a coverslip to facilitate the individual identification and counting of fluorescent cells. Fluorescent images were taken on a Zeiss AX1 Epifluorescence microscope and analyzed in ImageJ.

Outgrowth assays were performed as described by Hannan et al.^46^ with the following modifications. Microfluidic devices were treated with 10 μg/ml fibronectin in DPBS solution and incubated under laminar flow to air dry, uncovered, at room temperature for 30 min. Fibronectin‐coated devices were then washed 2× with 40 μl sterile PBS and 2× with 40 μl KSOMaa culture medium before priming with KSOMaa as normal. Dishes were pre‐equilibrated at 37°C under 5% CO_2_, 5% O_2_ in humidified nitrogen for 2 h before use. Embryos were cultured in these conditions throughout early cleavage and blastocyst development. Embryos were checked daily for developmental progression on a Nikon inverted microscope. Blastocyst development was recorded on day 5, while culture was extended by a further 96 h for attachment and outgrowth formation.^46^ Outgrowth was imaged and diameters measured and recorded at 200× power using RI viewer software. At the end of outgrowth culture, all embryos were tested for attachment by gentle pipetting using a 170 μm embryo pipette. Embryos which were not displaced by the flow of medium were considered to be attached.

### 2.5 Experiment 2: Blastocyst rates, energy substrate consumption and mitochondrial polarization

To define the loading capacity of device culture, groups of 10, 20, 30 and 40 2‐cell mouse embryos were cultured to the blastocyst stage in microfluidic devices in parallel to controls (10 embryos in one 10 μl culture microdrop) before metabolic profiling. A total of 220 blastocysts were analyzed in each treatment group from five replicate cultures. Therefore, the embryo: medium ratio varied from the widely used 1:1 μl in microdrop controls to 1:0.01 in the chamber of the 40 group.

Glucose and pyruvate consumption were measured using spent media from devices or microdrops by the method of Guerif et al.^28^ and expressed as mean pmol/embryo/h ± SEM. Briefly, 10 μl of reaction mixture was added to the base of a black 384 well microplate. All media from each device was collected in a 500 μl microtube and measured with a pipette to identify and account for any change in total volume. One microliter of spent media from each device or microdrop was added to each reaction mixture well and the difference in NAD, NADH or NADP signal, measured at 340/460 nm, was calculated. Concentrations were calculated using a 6‐point standard curve after correction for the final volume retrieved from each device. Consumption data were expressed in terms of pmol/embryo/h using the recorded culture time.

Day 5 blastocysts were labeled and imaged within devices using the ratiometric mitochondrial dye JC‐10 (Molecular Probes). Briefly, a 1 mg/ml JC‐10 stock solution was prepared in KSOM culture media. JC‐10 stock was diluted to 10 μg/ml in pre‐equilibrated M2 media (Millipore). Following removal of spent media for energy substrate assays, media was replaced with this JC‐10 solution and incubated at 37°C for 30 min. Control embryos must be moved to glass slides for imaging, while device embryos were imaged within devices. Imaging was performed on a LSM 780 confocal microscope, while image analysis was carried out in ImageJ. JC‐10 accumulates in the matrix of polarized mitochondria, forming J‐aggregates with punctate red fluorescent signal. In areas of reduced mitochondrial polarization, the dye tends to remain in monomeric form with diffuse, green signal. A ratio of red+green/total signal intensity, thus indicates overall mitochondrial polarization in the images sample and is termed the mitochondrial polarization ratio.^47^ Data were represented as red fluorescence/total red + green fluorescence to account for any nonspecific fluorescence across both channels. Higher values indicate a higher level of mitochondrial polarization throughout the measured region of interest.

### 2.6 Experiment 3: Real‐time PCR (qPCR) of single blastocysts

Groups of 10, 1‐cell murine embryos were cultured in KSOM medium in 10 μl microdrops (control) and inside the devices. Ninety blastocysts were used in each treatment group from three replicate cultures. From these cultures, 10 stage matched, expanded blastocysts were selected and analyzed for each experimental group. Individual blastocysts were recovered from devices or control microdrops, and immediately transferred into 2 μl RNAGEM lysis buffer (RNAGEM Tissue Plus®, MicroGem International PLC, Southampton, UK) and frozen at −80°C. For the construction of cDNA libraries of individual blastocysts, we modified an existing protocol.^48^ In summary, total RNA from single blastocysts was isolated using an RNAGEM‐extraction reagent mastermix. The total RNA was reverse‐transcribed to cDNA using a first strand cDNA synthesis kit (Thermo Fisher Scientific Inc., UK). Quantitative PCR (qPCR) was performed on 6‐fold diluted sample cDNA to analyze expression of 53 selected genes associated with blastocyst development and cell differentiation. Accession number, primer sequence and product length of target genes are presented in Table S1. Ten individual blastocysts were analyzed in each experimental group. mRNA expression was examined using SYBR Green Master PCR mastermix (Thermo Fisher Scientific Inc., UK) with an ABI 7500 RT‐PCR System (Applied Biosystems) over 40 cycles and using the house keeping genes listed in Table 1. Data were analyzed with 7500 Software using relative quantification analysis.

**TABLE 1:** Summary of gene symbol, accession number, product length and primer sequences of the housekeeping genes

Symbol	Name	Accession	Size (bp)	Sequence of nucleotides (5′⟶3′)	
Eif1	Eukaryotic translation initiation factor 1	NM_011508	186	Forward	AAGGGCTACCTTTCCAGAGA
				Reverse	GCACTGGCTCGTACTGAGTT
Rpl13a	Ribosomal protein L13A	BC086896	215	Forward	ATGACAAGAAAAAGCGGATG
				Reverse	CTTTTCTGCCTGTTTCCGTA
Gapdh	Glyceraldehyde‐3‐phosphate dehydrogenase	BC083080.1	223	Forward	CTGGAGAAACCTGCCAAGTA
				Reverse	TGTTGCTGTAGCCGTATTCA
Rplp0	Ribosomal protein, large, P0	NM_007475	202	Forward	AACCCAGCTCTGGAGAAACT
				Reverse	GGAAGAAGGAGGTCTTCTCG
Ywhaz	Tyrosine 3‐monooxygenase/tryptophan 5‐monooxygenase activation protein, zeta polypeptide	NM_011740	181	Forward	AGCAGGCAGAGCGATATGAT
				Reverse	TTCTCAGCACCTTCCGTCTT
Actb	Actin, beta	NM_007393	160	Forward	AAGAGCTATGAGCTGCCTGA
				Reverse	TACGGATGTCAACGTCACAC
18 s	18 s ribosomal RNA	NR_003278	298	Forward	ATTCCGATAACGAACGAGACT
				Reverse	AGCTTATGACCCGCACTTACT
Pgk1	Phosphoglycerate kinase 1	NM_008828	185	Forward	GCAGATTGTTTGGAATGGTC
				Reverse	TGCTCACATGGCTGACTTTA

### 2.7 Experiment 4: Global untargeted metabolomics

To investigate impact of microfluidic culture on the embryo secretome and to establish if the PDMS matrix released low molecular weight species or sequestered hydrophobic biomolecules from culture media,^49^ we analyzed and compared samples of spent media (KSOM) collected from devices to samples of spent media collected from control KSOM microdrops cultured with and without embryos using global untargeted metabolomics. Despite the additionally complexity, standard KSOM in vitro culture media was used rather than a basic salt solution, for example, PBS to ensure data were relevant to in vitro culture practices. 1‐c murine embryos were cultured using the same embryo‐to‐volume ratio in microdrops or PDMS devices. Specifically, groups of 10, IC zygotes were cultured in either 40 μl KSOM microdrops under oil or in each microfluidic device, where 20 μl drops of media were added to each inlet and outlet ports. Samples of spent media (40 μl) were collected from devices or microdrops to assess preimplantation embryo metabolomics. Importantly, to allow stage‐matched comparison of embryo metabolite production/consumption, samples were collected when embryo development had progressed to the fully expanded blastocyst stage. Specific to this study, the blastocyst stage was achieved at day 4 in microdrops and day 5 in devices. Compared to traditional microdrop culture, in this device there is in fact a slight deviation in time (between 12 and 24 h) for embryos to reach the blastocyst stage. For this reason, in this work samples from devices are collected after 5 days of culture and samples from microdrops after 4 days of culture. Equal media volumes from the two culture platforms were considered in order to maintain a constant embryo to media ratio. We assumed that the metabolite content measured in culture media drops is representative of that in the culture chamber, since slow diffusion phenomena induce transport of biomolecules along the channels to the media drops.

As a control, spent media from devices without embryos was compared to media collected from microdrops without embryos using the same incubation time performed for embryo culture. These experiments were performed to investigate PDMS leaching and/or molecule absorption and adsorption. Spent culture media was frozen and stored at −80°C prior to sample preparation for analysis. Culture media samples (100 μl) were thawed on ice and prepared using previously described methods. Briefly, 300 μl of dry ice cooled methanol was added to individual culture medium samples and incubated overnight at −80°C. Individual samples were spun down to remove proteins and the subsequent supernatant was used for analyses. Samples were separated and analyzed using reverse‐phase liquid chromatography connected to a Thermo Scientific Q Exactive HF (LC‐Hybrid Quadrupole‐Orbitrap MS/MS) instrument using positive ion mode MS.^50^, ^51^, ^52^ MS raw data were imported, processed, normalized, and reviewed using Progenesis QI v.2.1 (Non‐linear Dynamics, Newcastle, UK). Resulting MS data were utilized for relative quantitation. The full collection of raw data has been published on Metabolomics Workbench.^53^ Tentative and putative annotations^54^ were determined using accurate mass measurements (<5 ppm error), isotope distribution similarity, and manual assessment of fragmentation spectrum matching from the human metabolome database,^55^ Metlin,^56^ MassBank,^57^ and the National Institute of Standards and Technology database.^58^ Increased confidence in the annotation of many features was achieved by manually assessing spectral match and RT consistencies between experimental data and chemical standards within a curated in‐house library.

## 3 STATISTICAL ANALYSIS

Data were analyzed using GraphPad Prism 8 software (Graph Pad Software Inc., California, USA). All data sets were first tested for fit to the normal distribution by D'Agostino‐Pearson test for normality. In experiment 1, all normal data sets were compared by Student's *t*‐test, while all nonparametric data were compared by Mann–Whitney *U* test. In experiment 2, all data sets were nonparametric and therefore tested for significant differences between groups by the Kruskal–Wallis test with post‐hoc Dunn's test for multiple comparisons. In all instances, significance was determined as *p* < 0.05. For metabolic activity experiments, results were checked for statistical differences between groups by ANOVA with post‐hoc Bonferroni test. qPCR results were analyzed by the comparative threshold cycle (*C*_t_) method. Relative expression ratios were obtained using as internal control the mean of *C*_t_ values of eight housekeeping genes from the sample of interest. Student *t*‐test statistics was used to compare gene expression levels between samples from the different groups. Data were considered to be statistically different with a *p* value of <0.05. The values presented are mean ± SEM for the numbers of samples/replicate cultures shown.

For MS, compounds with <20% coefficient of variance (%CV) were retained for further analysis. Within Progenesis QI, a one‐way analysis of variance (ANOVA) test was used to assess significance between groups and returned a *p*‐value for each feature (retention time_m/z descriptor), with a nominal *p*‐value ≤0.05 required for significance. Significant features were further filtered using a fold change threshold ≥ |2| deemed as significant.

## 4 RESULTS

### 4.1 Microfluidic design is compatible with standard embryo culture methods

**FIGURE 2:**
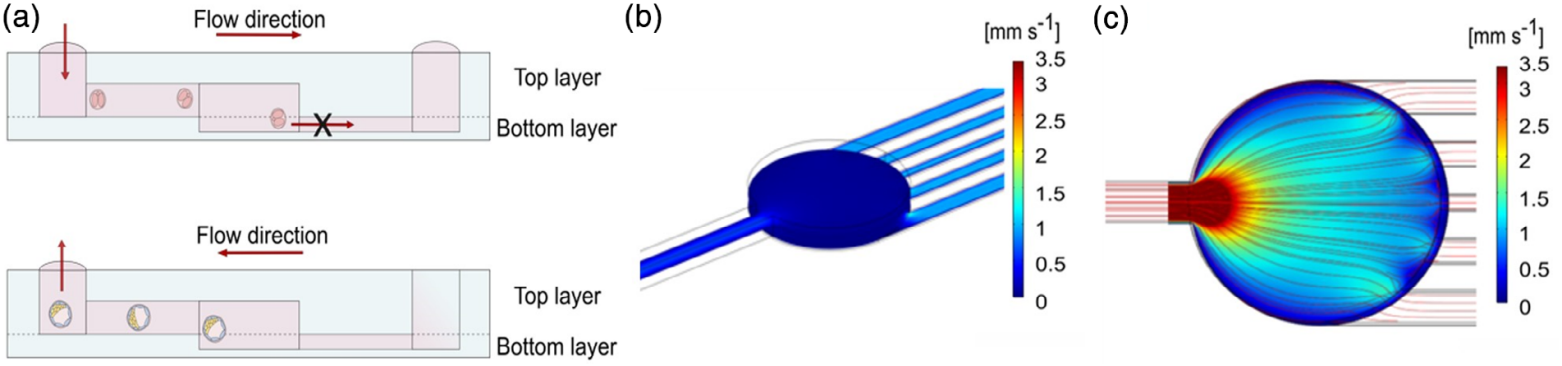
Microfluidic device design. (a) Embryos are introduced from the inlet port in the culture chamber through the inlet channel. Being the embryos diameter bigger than the outlet channels, they remain trapped in the chamber. Once developed, embryos are aspirated from the inlet port. (b) The Finite Element Model of the average velocity shows an increase in the lateral channels compared to the main chamber owing to their small dimensions. (c) Fluid flow analysis of velocity magnitude surface plot shows the velocity field generated in the fluidic systems

The microfluidic device presents two fluidic ports connected to a culture chamber by two microfluidic channels (Figure 2a–c). The volume of medium in the chamber is 400 nl, thus significantly reduced compared to the traditional 5–50 μl used in microdrop technique and the volume limit (1.5–2 μl) used in the ultra microdrop method. This reduction of volumes is typical of microfluidic dimensions, and other groups have pushed this approach even further.^19^ The inlet and outlet channels are at different heights and positioned in two different layers (top and bottom): the single inlet channel is 200 μm in height and 250 μm in width. The five narrow outlet channels are at lower level and 30 μm tall, thus smaller than the size of a mouse embryo (diameter ~60 μm). These dimensions allow the embryos to be loaded and to reach the chamber, where they cannot flow any further (see Video S1). Embryos were left undisturbed during development to blastocyst stage (diameter ~100 μm, 3 days) and then aspirated back through the inlet channels to proceed with further molecular analysis or potential embryo transfer procedures. The final device, measuring 4 by 4 cm, can be placed in a standard 60 mm IVF culture dish, sterilized and used in a MINC™ Mini incubator (Figure 1b).

### 4.2 Minimal stress or damage observed by flow and shear stress during loading and through the development process in the microfluidic device

The design of the device allows for the capture of the embryos in the middle chamber owing to the increased hydraulic resistance encountered when reaching the chamber (Figure 2a). Furthermore, the wall shear stress generated within the channels during loading and retrieval reaches maximum values of 0.17 dyn cm^−2^, which is seven times lower than values presented in literature (1.2 dyn cm^−2^ shear stress caused lethality within 12 h for E3.5 blastocysts^44^, Figure S1).

The wide culture chamber and its consequent lower hydraulic resistance^59^ ensure the trapping of the embryos within the culture chamber. During fluid loading, the velocity profile is significantly reduced from the inlet channel (hydraulic resistance in the order of) into the culture chamber due to an increase in hydraulic resistance (from ~10^10^ to ~10^–12 to 13^ (Pa s)/m). The inlet and outlet channels have similar fluid velocity profiles. This particular design that includes five narrow outlet channels instead of a single outlet channel, as wide as the culture chamber diameter, was adopted (i) to optimize the flow profile within the culture chamber (Figure S2) and (ii) to observe fabrication requirements preventing the PDMS structure from collapse.

An additional design criterion was used to favor the spreading out of the embryos across the whole culture chamber and to ensure a homogeneous perfusion of medium in the chamber. As shown in Figure 2c and in Figure S2, a wide velocity profile of the optimized device design was obtained within the culture chamber along the cross‐sectional direction when the device included multiple channels disposed parallel to the inlet channel. Real flow characterization performed by injecting fluorescein in the microfluidic chamber, showed that the green dye diffused and reaches equilibrium in 8 s, as reported in Figure S2.

As predicted from the computational analysis, the velocity profile along the cross‐sectional direction of the culture chamber is wide as the chamber width (Figure 2c and Figure S2). Considering the optimal diffusion of molecules and nutrients within the chamber (e.g., fluorescein) that this can provide, and the transient mechanical stimulation exerted on the embryos, these results suggest that embryos homogeneously spread in the chamber and receive equal amount of nutrients. Maintaining the embryos un‐clustered but at close reciprocal distance favors paracrine signaling, local accumulations of growth factors secreted by the embryos and the formation of gradients of nutrients in the embryo environment, as found in vivo.

After thawing and washing with fresh medium, 1‐cell mouse zygotes were loaded in the device using a 145 μm pipette. These pipettes are traditionally used for embryo culture and has a tip size compatible with the inlet port of the device. The embryos are manually injected into the inlet port and owing to fluid movement they safely move through the inlet channels and reach the central chamber (Movie S1).

The microfluidic device was designed to minimize the clustering of embryos, even when cultured in larger groups of 20, 30 and 40 embryos.

### 4.3 Experiment 1: Blastocyst rate, hatching and outgrowth were not affected by the microfluidic environment

**FIGURE 3:**
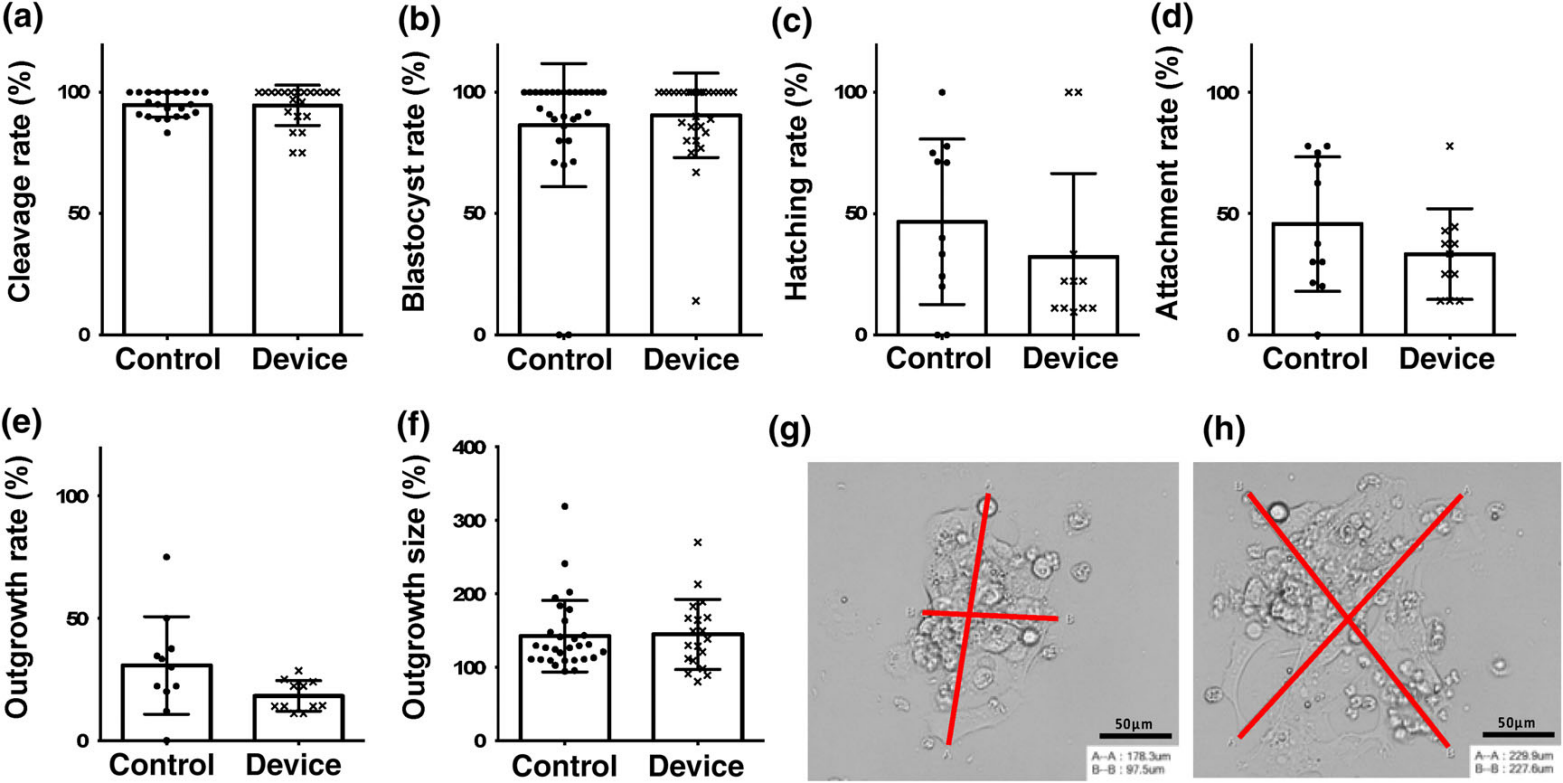
Comparison of preimplantation embryo development, data from experiment 1 in which embryos were cultured in groups of 10. (a) Cleavage rates (94.62 ± 8.3%, *n* = 30 vs. control 94.84 ± 5.1%, *n* = 30, *p* = 0.48). (b) Day 5 blastocyst rates (out of total embryos cultured) (90.48 ± 17.4%, *n* = 30 vs. control 86.40 ± 25.4%, *n* = 30, *p* = 0.6), (c) Hatching rates (out of total blastocysts) (32.18 ± 34.3%, *n* = 30 vs. control 46.61 ± 34.1%, *n* = 30, *p* = 0.32), (d) Attachment rates (out of total blastocysts) (33.22 ± 18.7%, *n* = 11 vs. control 45.64 ± 27.7%, *n* = 11, *p* = 0.35), (e) Outgrowth rates (out of total blastocysts) (18.2 ± 6.2%, *n* = 11 vs. control 30.6 ± 19.9%, *n* = 11, *p* = 0.08), (f) Mean outgrowth size (μm) (144.5 ± 47.8 μm, *n* = 19 vs. control 142.1 ± 48.7 μm, *n* = 19, *p* = 0.72). Values plotted are means ± SD for the number of embryos. (g, h) Representative images of blastocyst attachment and outgrowth by embryos cultured in fibronectin‐coated microdrops and microfluidic devices respectively as an index of implantation potential: red lines indicate outgrowth diameter measurement bars created in RI viewer (A‐A and B‐B). Scale bar 50 μm

Assessment of morphology at distinct time points is regularly used for evaluating embryos' quality. Early cleavage, occurring on average at 24 h after pronuclear fusion, has been correlated with embryo quality together with blastocyst rate and hatching rate both in mice and in human. Cryopreserved zygotes were thawed and cultured in KSOM in groups of 10 in standard 10 μl drops and in microfluidic devices. As summarized in Figure 3a–h, embryo development during microfluidic device culture was not altered when compared to traditional microdrop culture. Specifically, no significant differences were observed in cleavage rate, blastocyst rate or hatching rate (Figure S3).

Blastocyst adhesion competence in vitro was quantified by transferring embryos into fibronectin‐coated plates for analysis of attachment and outgrowth formation (Figure 3e–h). This assay mimics the natural mechanism of adhesion of the blastocyst trophoblast cells onto the endometrium which is regulated by interaction between the integrins naturally expressed on endometrial cells and on the apical surface of competent blastocysts.^60^, ^61^ Assessing the extension and adhesion of the hatched blastocysts is used as an index of their implantation potential. The blastocyst attachment data were similar between embryos grown in microfluidic device cultures (33.22 ± 18.7%, *n* = 11 replicate cultures) and microdrop cultures (45.64 ± 27.7%, *n* = 11 replicate cultures); these results were not statistically significant different from each other (*p* = 0.35). Outgrowth rate, defined as the percentage of blastocysts forming outgrowths (18.2 ± 6.2%, *n* = 11 vs. control 30.6 ± 19.9%, *n* = 11 replicate cultures, *p* = 0.10) and mean diameter of the outgrowths formed were also not statistically different (144.5 ± 47.8 μm, *n* = 19 vs. control 142.1 ± 48.7 μm, *n* = 19, *p* = 0.72).

**FIGURE 4:**
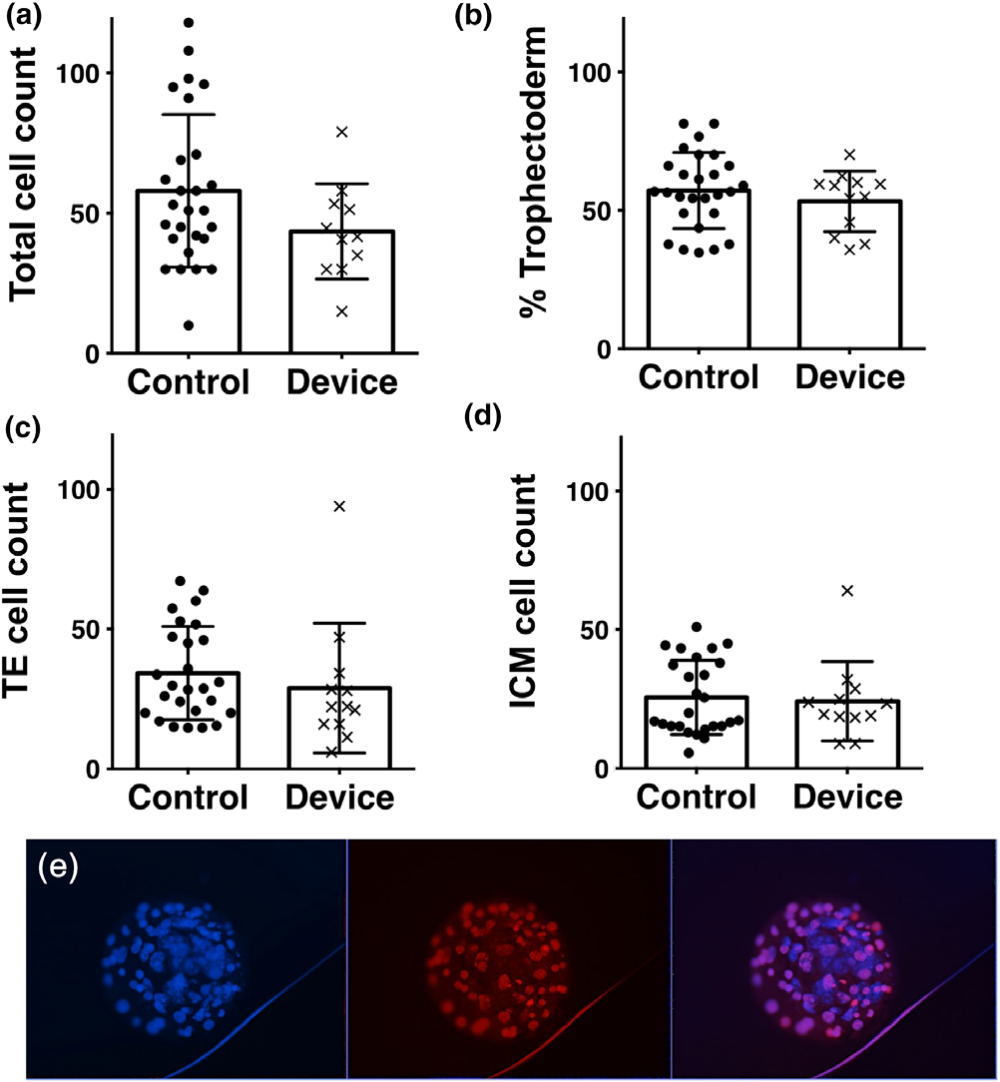
(a) Total cell count 44 ± 17.0 cells, *n* = 12 embryos, *p* = 0.1 versus control 58 ± 27.2 cells, *n* = 27 embryos. (b) Cell allocation ratio (% Trophectoderm) 53.3 ± 10.9%TE, *n* = 12, *p* = 0.4 versus control 57.2 ± 13.7%TE, *n* = 27, *p* = 0.4. (c) Trophectoderm (TE) cell count 28.9 ± 23.2, *n* = 12, versus control 34.2 ± 16.7, *n* = 27, *p* = 0.42. (d) Inner Cell Mass (ICM) cell count 24.2 ± 14.3, *n* = 12, versus control 25.6 ± 13.4, *n* = 27, *p* = 0.77. Values plotted are means ± SD for the number of embryos analyzed. (e) Representative conventional epifluorescent image of a blastocyst within the device stained with Hoechst 3342 (all cells) and propidium iodide (trophectoderm only) and imaged in the 460 nm (blue) and 560 nm (red) channels respectively. Scale bar 50 μm. Brightness and contrast digitally enhanced for clarity of presentation

Cell number in a preimplantation embryo is directly correlated to the embryo health, developmental potential and ability for cell cycle progression. Total cell counts and cell allocation ratios in Day 5 blastocysts was carried out with an antibody‐free differential staining method (Figure 4a–c).^47^ Total cell numbers were similar between controls and devices (Figure 4a). There were also no significant differences between cell allocation to trophectoderm or inner cell mass lineages as expressed as percentage trophectoderm of total cells (Figure 4b). Representative images are displayed in Figure 4c. The similar allocation and formation of the extra‐embryonic cell lineage (trophectoderm) and the ICM supports the negligible effect of the microfluidic confinement on embryo potency.

### 4.4 Experiment 2: Embryo group size limits development and metabolic activity in the microfluidic chamber

The number of embryos cultured in a single microdrop and the volume of the microdrop are two variables that affect embryo development during culture. Different protocols have been compared to identify an optimal range of embryo density, in terms of μl of medium available per embryo. In a microfluidic system, the embryos are confined in a different environment, specifically a 400 nl static chamber is linked through two side channels and allows two 10 μl drops of medium to be available to provide nutrients during ~3 days of culture. It is extremely important to evaluate how the different fluidic environment can support embryo growth and provide sufficient nutrients.

**FIGURE 5:**
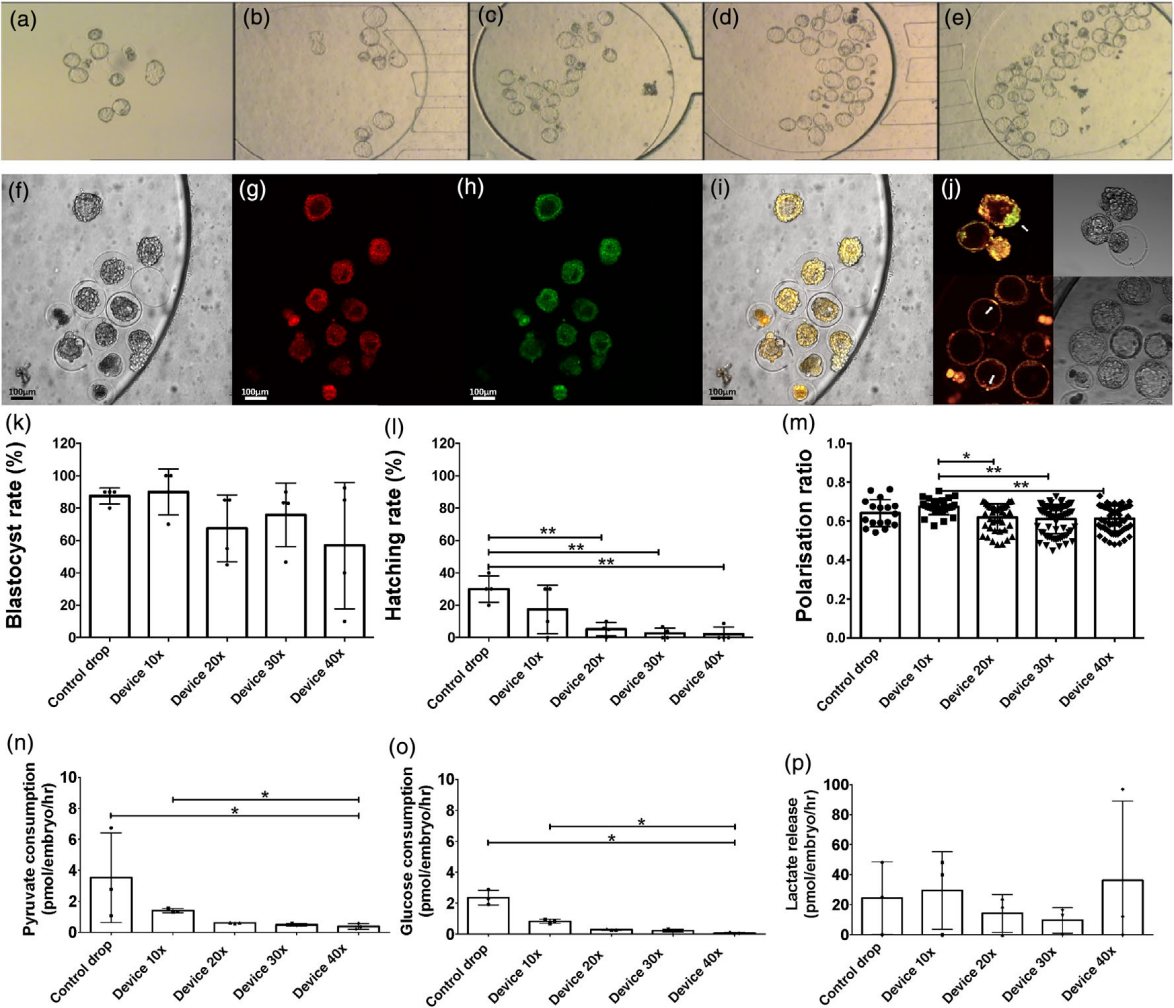
Loading capacity experiment, data from experiment in which embryos were cultured in groups of 10–40. (a–e) Representative bright field images of Day 5 blastocysts in microdrop and device culture groups: (a) microdrop controls, (b) 10× in device, (c) 20× in device, (d) 30× in device and (e) 40× in device. (f–i) Representative conventional epifluorescent images of blastocysts (Device 20× group, 11 embryos in field of view) stained with the potentiometric mitochondria‐specific stain JC‐10 and imaged in the rhodamine isothiocyanate (RITC, b) and fluorescein isothiocyanate (FITC, c) channels respectively without removal from the device. Scale bar 100 μm. (f) Brightfield, (g) Red JC‐10 staining indicating regions of high mitochondrial polarization, (h) Green JC‐10 staining indicating lower mitochondrial polarization, (i) Merged image. (j) Further representative images of JC‐10 labeled blastocysts, with inner cell mass indicated by white arrows. (k) Blastocyst rate (*n* = 4 cultures). (l) Hatching rate (*n* = 4 cultures). (m) Quantified blastocyst polarization ratio data (*n* = 4 cultures) show higher value when culturing groups of 10 embryos inside the device. (n–p) Energy substrate turnover (*n* = 4 cultures). Values plotted are means ± SD for the number of embryos analyzed. “*” Indicates significant differences at *p* < 0.05

When comparing groups of 10×, 20×, 30× and 40× embryos grown in the microfluidic device, due to the variability of the data the overall blastocyst rate, recorded on day 5, was not significantly different between groups of different size (*p* = 0.25) (Figure 5e). That said, blastocyst rates were highest in the device 10× group, and decreased with the size of the group, from ~90% in the 10× group down to ~56% for the larger group of 40× embryos. Groups of 10× embryos very consistently gave 80%–90% blastocyst rates, while larger group sizes were very variable between replicate cultures. Hatching rate in devices significantly decreased compared to controls with increased group size (control group: 30 ± 8.2% compared to 40× group: 2.2 ± 4.4%, *n* = 4, *p* = 0.02, Figure 5f). This suggests that substrate competition outweighs increased paracrine effects in larger group sizes.

Development rates are a widely used key marker of ongoing developmental competence. However, to examine embryo competence in richer detail, metabolic parameters were investigated. Blastocysts were labeled and imaged directly within the devices using the ratiometric mitochondrial dye JC‐10 (Figure 5a–d,g) to evaluate changes in mitochondrial membrane potential, expressed as polarization ratio. Control blastocysts cultured in microdrops did not have a significantly different mitochondrial polarization ratio (0.64 ± 0.07, *p* > 0.92) compared to device‐cultured embryos, regardless of the group's size (Figure 5g). However, following device culture, blastocyst polarization ratio was significantly higher in 10× groups (0.67 ± 0.04, *n* = 27) compared to 20× groups (0.62 ± 0.07, *n* = 42, *p* = 0.01), 30× groups (0.61 ± 0.07, *n* = 71, *p* < 0.001) and 40× groups (0.61 ± 0.7, *n* = 66, *p* = 0.001) across four distinct repeat cultures. Overall blastocyst mitochondrial polarization ratio correlated strongly with blastocyst rate across all groups (*p* = 0.01), confirming the viable status of the embryos.

Control embryos had metabolic profiles typical of microdrop‐cultured murine blastocysts.^28^, ^29^, ^62^, ^63^ Device‐cultured embryo pyruvate and glucose consumption decreased with increasing group size (Figure 5h,i). Embryos cultured in groups of 40× had significantly reduced pyruvate (0.37 ± 0.2 pmol/embryo/h) and glucose consumption (0.07 ± 0.04 pmol/embryo/h) than controls (3.5 ± 2.9 pmol/embryo/h, and 2.4 ± 0.5 pmol/embryo/h, respectively, *p* = 0.02) or groups of 10× (1.4 ± 0.1 pmol/embryo/h, and 0.8 ± 0.1 pmol/embryo/h, respectively, *p* = 0.02) across four distinct repeat cultures. Pyruvate is the preferred energy substrate during early cleavage, while glucose consumption is low during early cleavage but tends to increase greatly with increased ATP generation through oxidative phosphorylation at the blastocyst stage.^29^ The present data suggest increased competition for these substrates within the more concentrated population of 40× embryos in comparison to groups of 10× in devices or control drops. Device embryos were seemingly more quiescent overall, with reduced variation between culture groups. The quiet hypothesis of Henry Leese suggested that embryos with moderate, or 'quiet', overall metabolism may be most viable.^64^, ^65^, ^66^ In contrast, embryos with insufficiently low metabolic rates and those with high metabolic rates may be stressed and have restricted development.^28^, ^29^, ^64^, ^65^, ^66^ For example, bovine embryos with an intermediate pyruvate consumption rate had a higher rate of blastocyst development.^28^ Embryos with metabolic profiles in the intermediate or *lagom* range and with a tighter distribution may be the most developmentally viable, due to undergoing less metabolic stress.^29^ It is tempting to speculate that with optimal group sizes, the minimal, physiologically accurate volume of available medium in device culture may encourage embryos toward this moderate metabolic profile and improve embryo developmental potential. Further studies will explore the impact of metabolic profile on development rate directly.

### 4.5 Experiment 3: Microfluidic confinement does not affect development and implantation related gene expression

**FIGURE 6:**
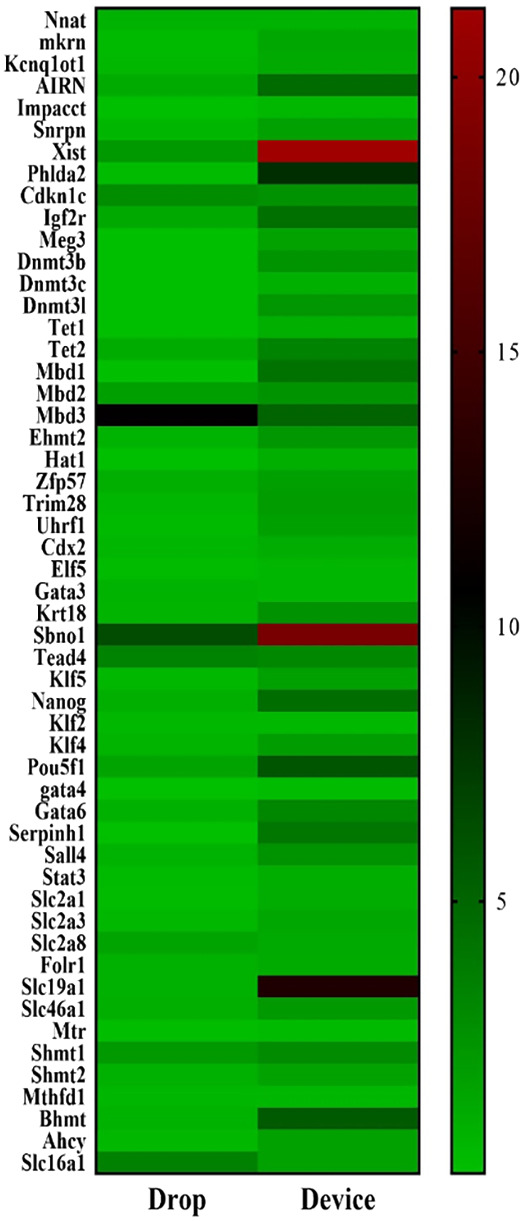
Heatmap representing gene expression of mouse stage matched, expanded blastocysts cultured for 4 to 5 days in the device compared to microdrop culture (*n* = 10 blastocysts for each treatment group, assayed individually). Scale: red indicates high expression and green is low expression

Using Real‐time PCR (qPCR) of single blastocysts, the expression of a panel of 53 genes involved in blastocyst development and cell differentiation was analyzed (Table S1). Overall, the gene expression profile of murine blastocysts cultured in the microfluidic device is similar to that observed in blastocysts cultured in traditional microdrops (Figure 6). Exceptions include the imprinted gene *Xist* and *Sbno1* which resulted highly expressed in the device group (*p* > 0.05) compared to the microdrops group. *Xist* is known to have a role in the X chromosome inactivation.^67^ Recent studies also provided evidence that absence of *Xist* can cause molecular defects in developmental pathways such as altered expression of extraembryonic‐development genes and pluripotency genes.^65^ Although it is still under debate how changes in *Xist* expression levels specifically impact embryo development, those results could implicate an effect of the microfluidic culture on initiation of early paternal X chromosome inactivation and cell differentiation. An analysis of sex‐specific gene expression will be carried out in future study to further understand the effect of the microfluidic culture environment on the embryo development.^68^ On the other hand, *Sbno1* ^69^ expression is fundamental for murine blastocyst development and involved in trophectoderm differentiation. However, whether this observed altered expression of *Xist* and *Sbno1* could be associated to beneficial effects of the microfluidic device on embryo development needs to be investigated by further analysis such as measurement of absolute gene expression levels at different time during development, immunohistochemistry analysis for localization and comparison with in vivo—produced embryos of a comparable stage. Additionally, differential expression of marker genes involved in trophectoderm differentiation (*Klf5*,^70^ *Cdx2*,^71^ *Tead4*,^38^ *Gata3*,^39^ *Elf5*,^39^ *Krt18* ^37^) or ICM/epiblast development (*Stat3*,^72^ *Nanog*,^36^, ^37^, ^73^ *Pou5f1*,^74^ *Sall4*,^74^ *Gata6* ^73^) was measured (Figure S4). No statistically significant differences were observed in these genes when blastocysts developed in the microfluidic device were compared to those cultured in traditional microdrops. These preliminary results are thus not sufficient to conclude that the overexpression of *Sbno1* and *Xist* are effectively linked to abnormal development of the embryos or to limited implantation. These results could be further validated by increasing the sample size (i.e., number of expanded blastocysts for each experimental group) and by exploring the effect of each specific genetic alteration on embryo function and development.

### 4.6 Experiment 4: PDMS alters the medium composition but does not induce drastic changes in the embryo metabolism

**FIGURE 7:**
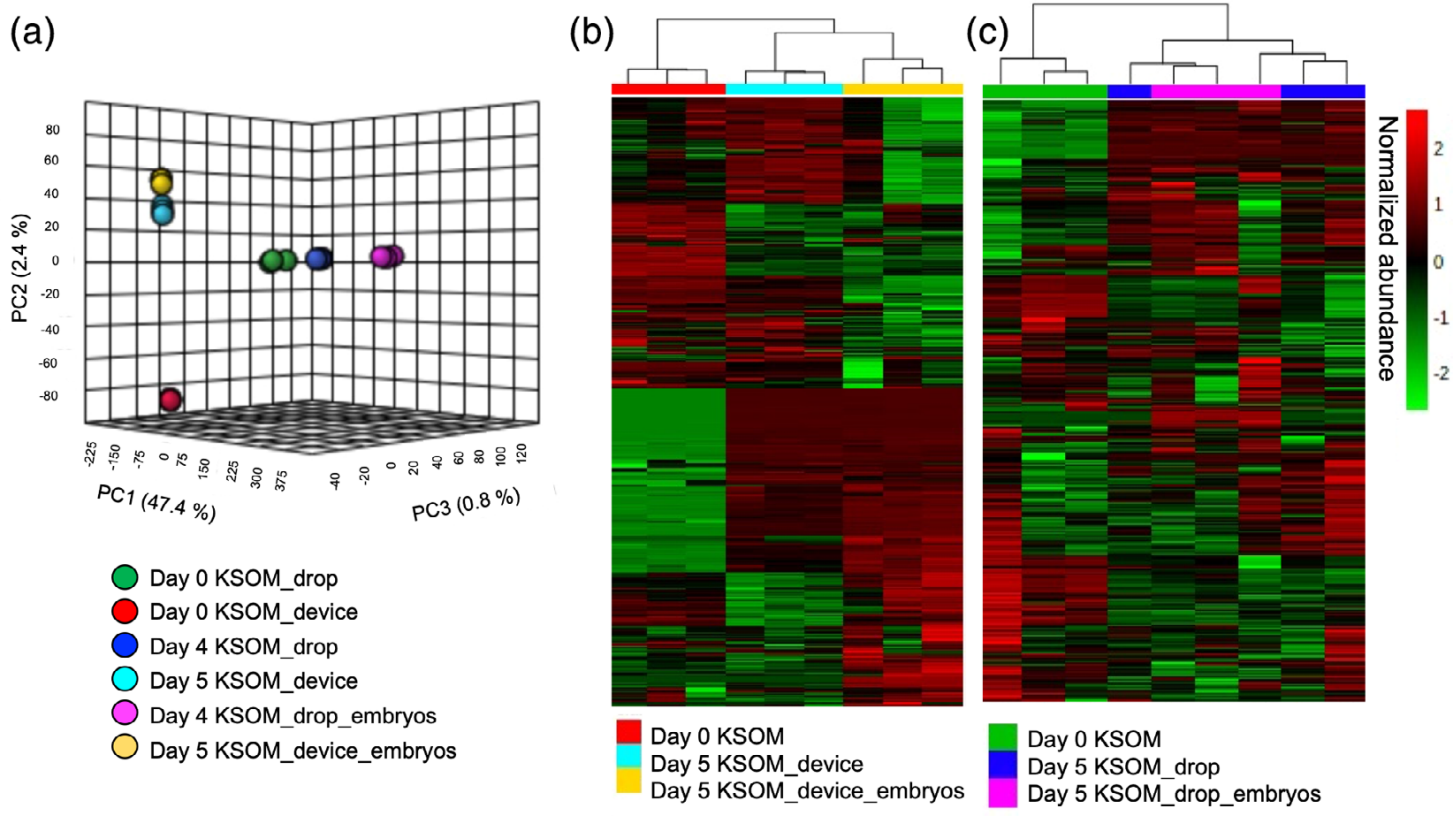
Principal component analysis (PCA) and heatmap visualization of global, untargeted mass spectrometry. (a) PCA plot of the LC–MS/MS data of medium samples collected after 4 days in microdrops or 5 days in devices and control KSOM (*n* = 3 replicate cultures of 10 embryos per experimental group). Heatmap analysis of media samples collected from (b) the microfluidic device or (c) control microdrop cultures with and without embryos. Sample replicates are visualized in columns column based on hierarchical clustering, with metabolites presented on individual rows. Species are colored based on normalized abundance from red (high) to green (low)

Global, untargeted liquid chromatography tandem mass spectrometry (LC–MS/MS) analyses were performed to compare spent medium (KSOM) on days 4 or 5 of embryo culture between each experimental group: KSOM, KSOM + microdrop, and KSOM + device. The metabolite composition of spent KSOM collected from the microfluidic device was compared with that of spent medium collected from control microdrops, both in the presence or absence of embryos (*n* = 3 replicate cultures of 10 embryos for each treatment group). The principal component analysis (Figure 7a) and heatmap (Figure 7b and Figure S4) show distinct clustering of the experimental groups. These global views revealed that while the majority of the detected metabolites were stable and present in abundance, there was a consistent subset of metabolites in culture medium with unique abundance profiles between device and microdrop culture methods.

**FIGURE 8:**
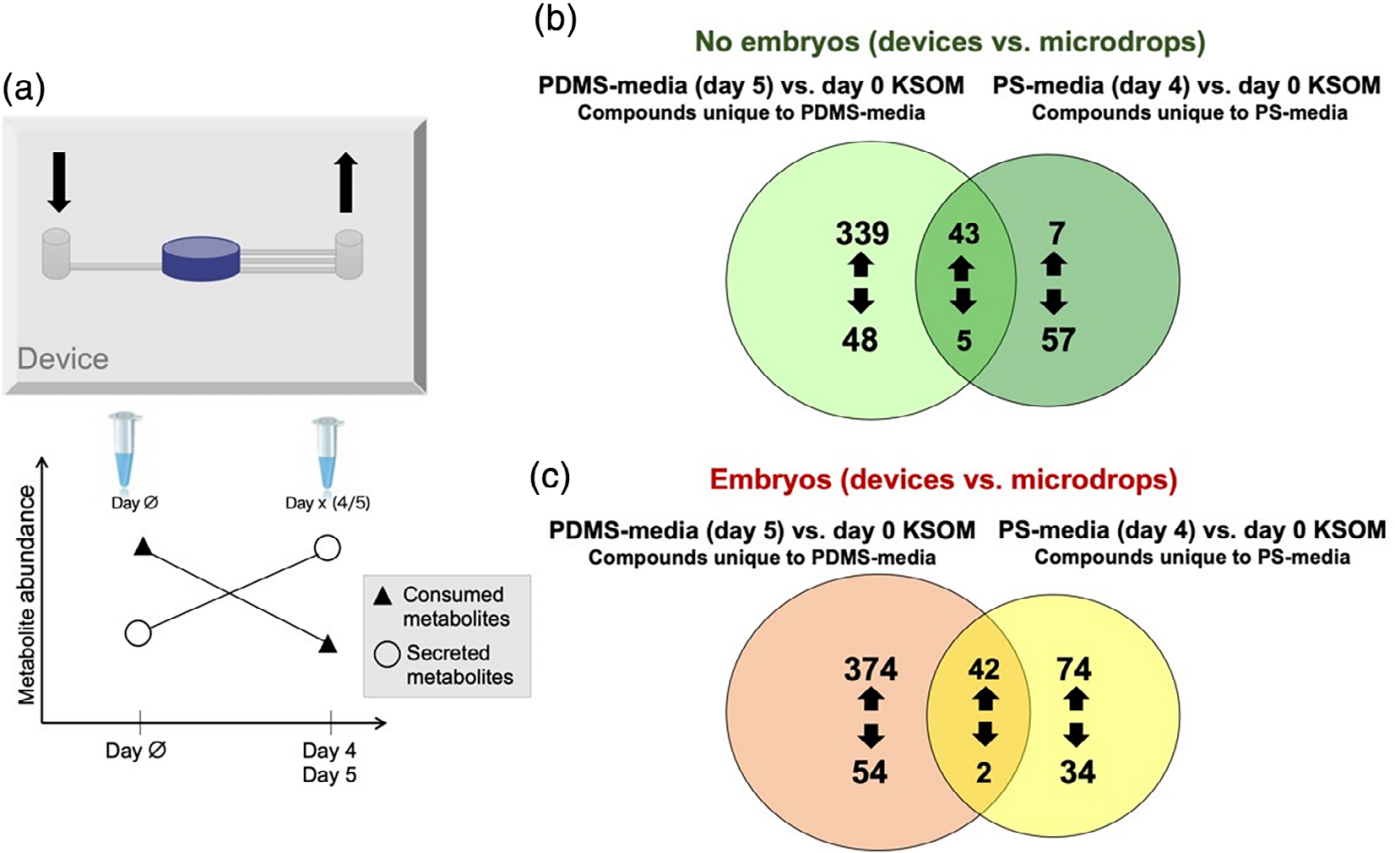
Venn diagram of dysregulated compounds in PDMS‐media and PS‐media compared to control (day 0 KSOM). (a) Schematic of the device with arrows indicating inlet (↓) and outlet (↑) ports (top). Changes in metabolite abundance in samples collected from microdrops (day 4) or devices (day 5) when compared to control. Increased and decreased compounds represent, respectively, released and consumed metabolites (bottom). (b) Comparison of dysregulated compounds in day 5 PDMS‐media and day 4 PS‐media without embryos. (c) Comparison of dysregulated compounds in day 5 embryo culture PDMS‐media and day 4 embryo culture PS‐media

Second‐order meta‐analysis of the individual pairwise comparisons (Figure 8a–c) allowed prioritization of endogenous or xenobiotic compounds (specific to PDMS, PS or mineral oil exposure) that were altered in abundance by the embryo culture method (device vs. microdrops). The Venn diagram shows shared and unique compounds for specific comparisons. A comparison of the compounds identified in spent media from devices (“PDMS‐media”) or spent media from control microdrops cultured in a standard Polystyrene dish (“PS‐media”) without the presence of embryos revealed a total of 547 compounds (Figure 8a). Using meta‐analysis, 48 compounds were common to the two groups (device vs. microdrop), whereas 387 were unique to the PDMS‐media group and 64 to the PS‐media group. Compounds unique to the device group, represent xenobiotic species directly associated with PDMS use. These data show 339 xenobiotic compounds were released by PDMS into the culture medium and 48 compounds were absorbed by‐ or adsorbed onto‐ the PDMS from the culture medium. The 339 species released into the culture medium, included detection of plasticizers such as butyl lactate, dimethyl sulfoxide, ethanol, 2‐[2‐(2‐butoxyethoxy)ethoxy]‐, *N*,*N*‐dimethylformamide, necatorine, pentaethylene glycol, triethylene glycol and tripropylene glycol. The effects of these potentially toxic compounds on embryo development must to be further investigated. The remaining compounds detected indicate breakdown products of media components produced by media degradation over 5 days of incubation at 37°C. These include peptides, amino acids and other small endogenous molecules (e.g., l‐glutamine, l‐tryptophan, *n*‐phenylacetylglutamic acid, pyridoxamine, dihydrolipoamide).

Similarly, among the 48 species that were significantly decreased in spent media from the device (Figure 8b), numerous biological compounds were identified that were sequestered by PDMS from the culture medium, these include: amino acids and dipeptides (e.g., isoleucyl‐isoleucine, isoleucyl‐leucine, isoleucyl‐phenylalanine, *n*‐acetyl‐l‐methionine, and valyl‐leucine).

Among the species that were significantly decreased (57 compounds) in spent media from microdrop culture, peptides and amino acids, such as l‐tyrosine, aspartylphenylalanine and phenylacetylglycine were identified. These data suggest that these molecules were absorbed or transformed by the plastic or mineral oil. The remaining detected compounds represent breakdown products of culture media that degraded over time.

Other organic species appeared down‐regulated in PS‐media and PDMS media suggesting a segregation of compounds from the medium both into the elastomer as well as in the plastic or the mineral oil used to prevent media evaporation. These molecules included tryptophol [xylosyl‐(1‐>6)‐glucoside], muramic acid, [3,5‐dihydroxy‐2‐(hydroxymethyl)‐6‐[3,5,7‐trihydroxy‐2‐(2,4,5‐trihydroxyphenyl)‐3,4‐dihydro‐2H‐1‐benzopyran‐6‐yl]oxan‐4‐yl]oxidane‐sulfonic, and 2‐methoxyestrone 3‐glucuronide.

Subsequently, a second‐order meta‐analysis of spent media collected with embryos present in the device and in the microdrop method was performed. Specifically, embryo cultured spent media from devices (day 5) were compared to embryo present spent media from microdrops (control, day 4) (Figure 8c). These analyses allow for us to determine metabolites and/or xenobiotics present in spent media after embryo culture (i.e., produced or consumed by the embryo during culture) as well as xenobiotics unique to device culture (i.e., associated with the fabrication process or released/absorbed by PDMS) or to control microdrop culture (i.e., associated with PS or the mineral oil used to prevent medium evaporation). The Venn diagram shows 428 species to only be present in embryo cultured PDMS‐media, 108 compounds were only observed in embryo culture PS‐media and 44 species were present in both embryo culture PDMS‐media and embryo culture PS‐media (Figure 8c).

**FIGURE 9:**
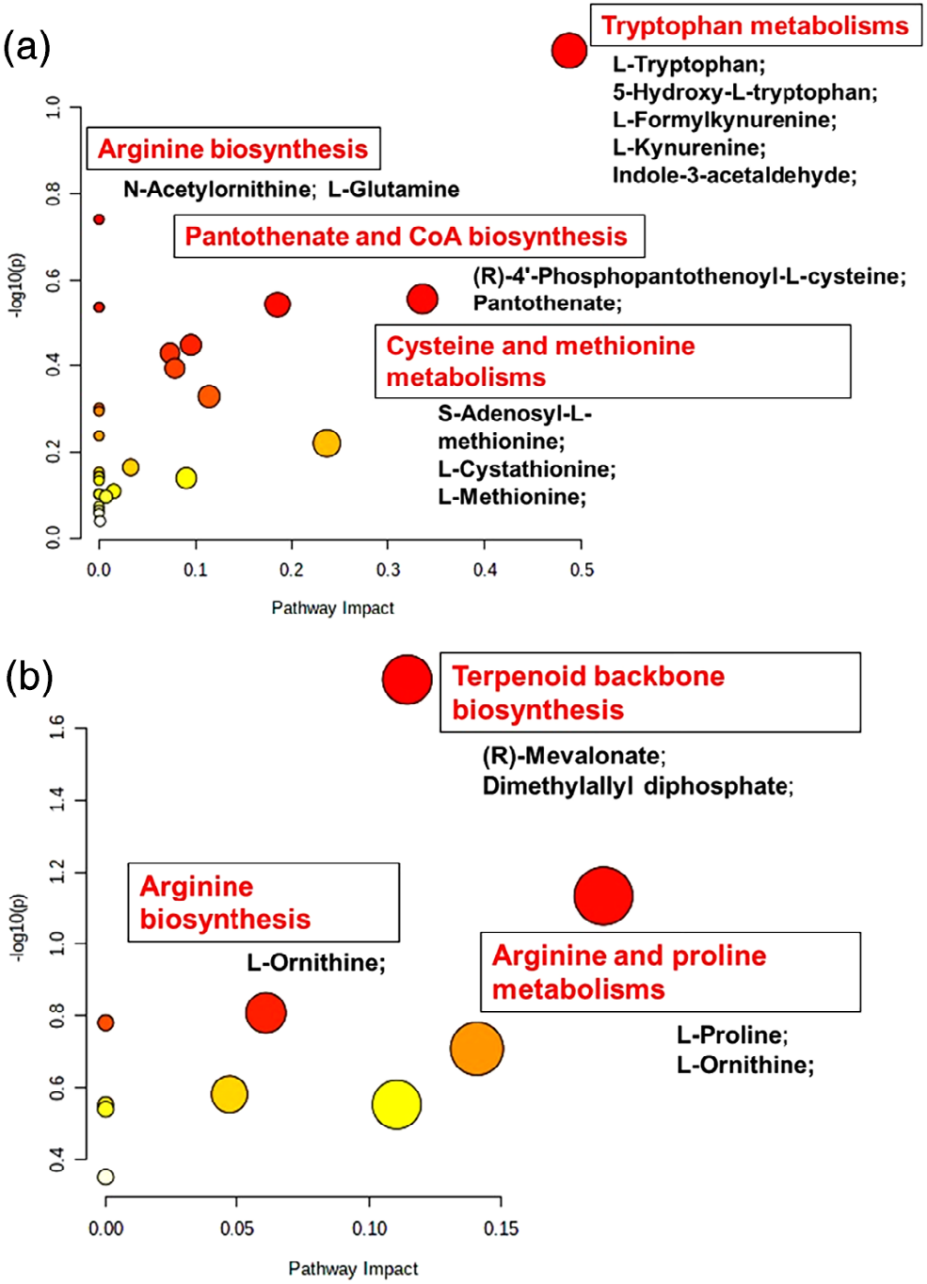
Pathways overrepresentation analysis. Summary of metabolic pathways of significant metabolites uniquely up‐regulated in day 5 embryo culture PDMS‐media (vs. day 0 KSOM) (a) and in day 4 embryo culture PS‐media (vs. day 0 KSOM) (b). The metabolome view contains results of the analysis generated by Metaboanalyst with all the matched pathways arranged by *p*‐values on Y‐axis, and pathway impact values on X‐axis

Putative metabolite identifications were used for pathway overrepresentation analysis using Metaboanalyst 4.0. From the comparison of the media from the device and the control culture in polystyrene dishes at the end of the culture, it was possible to identify 374 significant compounds uniquely up‐regulated in day 5 embryo culture PDMS‐media. The view map in Figure 9a revealed that the most significant enriched pathways for these metabolites were tryptophan metabolism, arginine biosynthesis, pantothenate and CoA biosynthesis, and cysteine and methionine metabolisms. Similarly, Figure 9b presents a list of the matched overrepresented pathways for the 74 significant compounds uniquely up‐regulated in day 4 embryo culture PS‐media. The metabolome view revealed that the most significant enriched pathways for these metabolites were terpenoid backbone metabolism, arginine biosynthesis, and arginine and proline metabolisms. Thus, the culture environment provided by the microfluidic system had a significant impact on some metabolic pathways (i.e., amino acid metabolism), although further assessment of its role on mouse embryo development and implantation potential need to be investigated.

The complete lists of metabolites used for the presented pathways overrepresentation analyses are provided in Tables S2 and S3.

Notably, among the 44 common metabolites present in both embryo culture PDMS‐media and embryo culture PS‐media, the relative abundance of 42 of these compounds were statistically significantly increased (*p* < 0.05 and fold change ratios >2) in both media when compared to control, these identified compounds include metabolic markers of preimplantation embryo development, such as pyroglutamic acid, 5′‐methylthioadenosine,^75^ ^,^ ^71^ hypoxanthine,^76^, ^77^ cytosine, *n*‐acetyl‐l‐methionine, and phenylacetylglycine. Preimplantation embryo development compounds represent metabolites produced by embryos in both culture conditions. Similarly, two compounds significantly decreased in spent embryo culture media compared to control (i.e., muramic acid and meticillin) might be consumed by embryos from the culture medium.

In summary, these results allow us to conlcude that metabolomic changes could be detected by mass spectrometric analysis in samples of media collected during embryo culture, and these were correlated with organic and inorganic compounds available to the embryos during their development in vitro.

## 5 DISCUSSION

Based on this extensive evaluation, we demonstrated the ability of our microfluidic system to enable culturing mouse preimplantation embryos, and that our microfluidic devices are compatible with generic lab equipment (such as optical microscopes, bench‐top incubators) and so are amenable to traditional embryo handling and analytical procedures (e.g., loading and retrieval with micropipettes) that are routinely used in ART as well as with research methods such as fluorescent staining and fixation.

The novel microfluidic device we have designed and fabricated in PDMS, provided culturing groups of murine embryos and enabled the use of several, noninvasive techniques for assessing embryo quality. The device was designed for optimal embryo loading of 10–12 embryos per device. The data indicated that the individual microfluidic compartments of the device permitted user friendly, efficient and reproducible loading and retrieval of the embryos. The microfluidic device and culture strategy was conducted in the absence of oil and served to demonstrate the compatibility of our device with standard bench top incubators and optical microscopes.

Mouse embryos grown in our microfluidic devices under optimized loading conditions demonstrated similar developmental capacity to embryos derived using the widely adopted microdrop culture method, with both cleavage and blastocyst rates exceeding 90% in all culture conditions. The evidence presented clearly demonstrates that if the optimal loading capacity of the device is exceeded then nutrient depletion and metabolic stress is induced which impacts severely on embryo health and developmental potential as indicated by the reduced capacity for microfluidic‐derived embryos to metabolize glucose and to hatch relative to controls. Under optimal loading conditions, microfluidic device culture had no impact on blastocyst cell number and blastomere partitioning between the ICM and TE. Furthermore, the blastocysts grown in microfluidic devices demonstrated equivalent capacity for hatching and out growth in vitro, factors which are indicative of implantation potential in vivo.^78^ Taken together, these data indicate that the novel microfluidic device presented here is embryo‐compatible and can successfully be used to support healthy embryo development to the blastocyst stage without the need for media changes or oil overlay.

The analysis of gene expression of single blastocysts derived in the microfluidic device provided a clear assessment of the influence of the microfluidic confinement on the embryo quality. The selection of genes correlated to the preimplantation and endometrial receptivity provides evidence to support limited alterations of the embryos during the 5 days of culture.

However, in this work gene expression levels were measured using relative quantification method, which did not allow to obtain information on the absolute expression value for each gene. Further analyses might use absolute quantification methods to measure expression levels of selected genes, to directly compare our findings with available gene expression datasets and elucidate specific impact of the gene alteration on embryo culture. Moreover, qPCR data will also be validated by increasing sample size, that is, the number of analyzed blastocysts for each experimental group, to provide further evidence on the impact of altered expression of key genes in embryo development and implantation potential. A particular focus will be addressed to metabolic and epigenetics markers to more deeply investigate the impact of microfluidic culture.

The combination of gene and metabolite data analyzes are complementary, metabolomics data allows us to identify at the molecular level metabolites released by the embryo or consumed by the embryo during development while genomic data allows us to follow changes of embryo development at the gene and protein level. Interestingly, the cultured medium is altered significantly at day 0, in accordance with previous work on PDMS absorption^49^ and the release of xenobiotic compounds in the medium is present in a few hours. These compounds can be ascribed to the unstable hydrophilic properties of the PDMS over time. These properties influence leaching and sequestration of small molecules by the elastomer.^79^ As revealed by the culture medium analysis, specific compounds can be found uniquely in the samples derived from the microfluidic system. In these data, low molecular weight species, which can be related to un‐crosslinked PDMS (molecular weight of dimethylsiloxane monomer = 74.15 Da), were detected in media samples extracted from the devices, confirming possible leaching of PDMS oligomer into the culture media. Second‐order meta‐analysis allowed for biologically important metabolite changes to be observed in the culture medium throughout embryo development. Pathways overrepresentation analysis showed that microfluidic culture had a significant impact on tryptophan metabolism pathway, which could explain the resulting activity of protein synthesis mechanisms fundamental during embryo development. Importantly, MS data did not reveal alteration of metabolites involved in metabolic pathways of glucose, pyruvate and lactate. This suggests that the microfluidic environment does not alter energy substrate metabolism, as also shown by our metabolic profile data (Figure 5h–j). Similarly, no significant changes in abundance of metabolites involved in oxidative stress processes were detected from the analyzed datasets, which demonstrates that the plastic does not impact the release of reactive oxygen species into the culture media. Identification and quantification of metabolite and xenobiotic compounds is not completed at this stage, however, these data show that the presence of the embryo in culture altered the composition of the medium in the device and in the microdrop method. These data also show that the number of common compounds in the different culture settings (device vs. microdrops) was changed. However, under optimal loading conditions the embryos developed successfully in the PDMS device, without significant alterations. The stability of PDMS may represent a challenge in the field of microfluidics and lab‐on‐chip research, however alternative plastics for manufacturing of the device could be used for this novel system. The methods used for evaluating the embryo development in this work should be performed at minimum for industrial manufacturing plastic. Importantly, from the morphokinetic analysis, these unexpected xenobiotic compounds observed in the medium did not induce significant alteration of the embryo development at different stages.

Oxygen availability is also a key requirement for embryo development.^80^ In this work, we assessed the effect of the confinement of the embryos in a close compartment, completely surrounded by PDMS, which is permeable to gas.^81^, ^82^ Oxygen tension of 5% for embryo culture has been widely adopted in both animal and clinical human IVF laboratories and considered a more physiological concentration that can boosts blastocyst development with no detectable adverse effects.^83^, ^84^ While it is true that the diffusion of oxygen through the PDMS could be modeled and compared to that through mineral oil and media, with optimal embryo loading we did not observe detrimental effects on the embryo development due to a different oxygen dynamic.

The microfluidic device culture facilitated harvesting of individual blastocysts for analysis of specific genetic profiles by real time qPCR. In this study the relative expression of 53 specific genes involved in blastocyst development and cell differentiation, trophoblast and epiblast development, aimed to exclude any genetic alteration induced by the microfluidic environment. In order to use the same technique and to correlate those specific genes to the blastocyst competence, a more consistent breeding protocol would be required, with standardization of fertilization method, sperm and egg donor and including different mouse strains.

The LC–MS/MS analysis of spent media revealed changes in the media composition, with sequestration of molecules and different consumption and release of compounds by the embryo in the microfluidic device and in the standard microdrop method. These data improve our understanding of the embryo metabolic activity and confirm the unstable characteristics of the prototyping elastomer. A more detailed analysis could be set up by collecting samples every 2 h to correlate altered media composition with abnormal embryo developmental rates to morula or blastocyst stage. Future analyses of the medium composition will be used to search for specific toxins, such as peroxides derived from the mineral oil, zinc, and other unknown contaminants that may be released into the medium from the oil or the plastic. Despite, the presented study provides valuable information of genetic and metabolic alterations induced in vitro by the culture environment, the presented data might be significantly improved by increasing sample size and number of experimental replicates. Importantly, further investigation are required to correlate the observed alteration in gene expression with metabolite changes and specific pathways affected by the different culture environment, with a particular interest on oxidative stress and metabolic activity.

Finally, the data presented show the feasibility of performing both PCR and metabolomics analyses from single stage matched blastocysts. The gene expression data generated from the microfluidic device shows the capability of profiling of preimplantation embryos in a microfluidic environment and confirms the impact microfluidic technology has in fundamental research in assistive reproductive technology. Further investigations are needed to explore the effects of device culture on embryo implantation and live birth rates. In this future study the precise morphokinetics of development in the devices, including quantitative data on morula) will be fundamental to correlate the birth rate with the preimplantation development. Embryo transfers in mice and assessment of birth rates will confirm the competence of embryos cultured in the microfluidic device and will allow to quantify time and costs saving introduced by this protocol. Further trials are now planned to evaluate the improvement in terms of birth rate, including different strains of the recipient mice, cryopreserved or fresh embryos and using specific surgical and not surgical protocols for embryo transfer.

This novel microfluidic technology eliminates the current standard use of mineral oil for embryo culture, thus reducing handling, failure, and costs for each culture set up. An entire group of 10–12 embryos can be rapidly loaded or retrieved in a single step, comparably to the microdrop technique. This represents an additional value in high throughout facilities to maximize the costs and the success of the culture. More importantly this new method also favors the adoption of NSET. Furthermore, the device characteristics can be revised and easily adapted to host larger animals (e.g., bovine, equine) and human embryos. This data set thus represents a first step toward to development of a new solution to improve the efficiency of human embryo culture during assisted conception treatment. The compatibility with the most advanced methods for assessing material toxicity and embryo quality will be key to correctly assess the safety and the performance of this new device.

## AUTHOR CONTRIBUTIONS

**Vanessa Mancini:** Data curation; formal analysis; investigation; software; validation; writing‐review & editing. **Paul McKeegan:** Data curation; formal analysis; investigation; methodology; software; validation; visualization. **Alexandra Schrimpe‐Rutledge:** Data curation; methodology; software; validation; visualization. **Simona Condreanu:** Conceptualization; data curation; formal analysis; investigation; methodology; software; visualization. **Stacy Sherrod:** Conceptualization; methodology; software; supervision; writing‐review & editing. **John McLean:** Conceptualization; funding acquisition; methodology; resources; writing‐review & editing. **Helen Picton:** Conceptualization; data curation; funding acquisition; methodology; resources; supervision; writing‐review & editing. **Virginia Pensabene:** Conceptualization; data curation; formal analysis; funding acquisition; investigation; methodology; project administration; resources; supervision; validation; visualization; writing ‐ original draft; writing‐review & editing.

## CONFLICT OF INTEREST

The authors do not have any conflict of interest with the described research results.

### PEER REVIEW

The peer review history for this article is available at https://publons.com/publon/10.1002/btpr.3194.

## Supporting information


**Appendix S1**: Supporting information.

## Data Availability

The data that support the findings of this study are available from the corresponding author upon reasonable request. The full collection of raw data on the MASS SPEC analysis has been published on Metabolomics Workbench at https://doi.org/10.21228/M8W99G.
