# Supplementary material for: Probing morphological, genetic and metabolomic changes of in vitro embryo development in a microfluidic device
Source: Biotechnol Prog. 2021 Jul 29;37(6):e3194. doi: 10.1002/btpr.3194 (PMC11475506; doi:10.1002/btpr.3194)
Supplement: Supplementary file 1 — Appendix S1: Supporting information. [file BTPR-37-e3194-s001.docx]

**Probing morphological, genetic and metabolomic changes of in vitro embryo development in a microfluidic device**

**V. Mancini and P.J McKeegan, A.C. Rutledge, S.G. Codreanu, S.D. Sherrod, J.A. McLean, H.M Picton, V. Pensabene**

**Supplementary material**

**Figure S1**


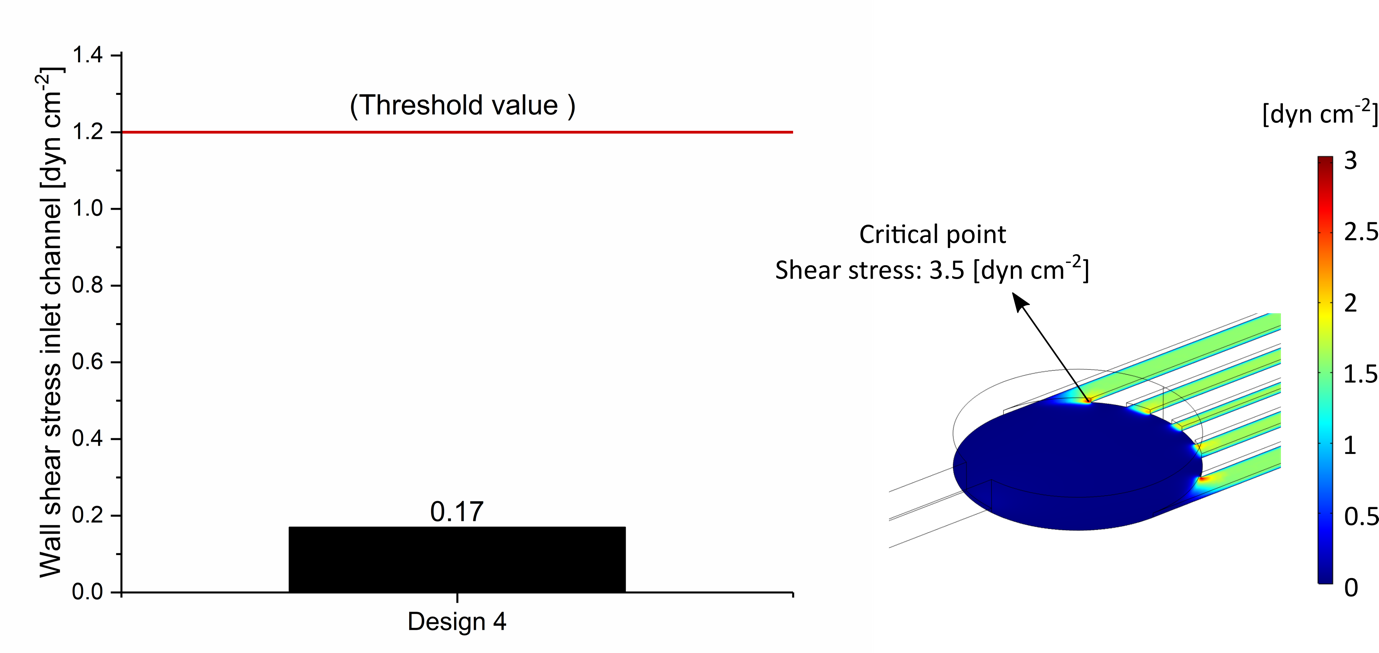


**Figure S1**. a) Computationally estimated inlet channels wall shear stress for the microfluidic device in its final version (Design 4), and b) fluid flow computational model of shear stress field surface plot during manual loading. The colour spectrum bar shows the shear stress field generated in the fluid systems.

**Figure S2**

**
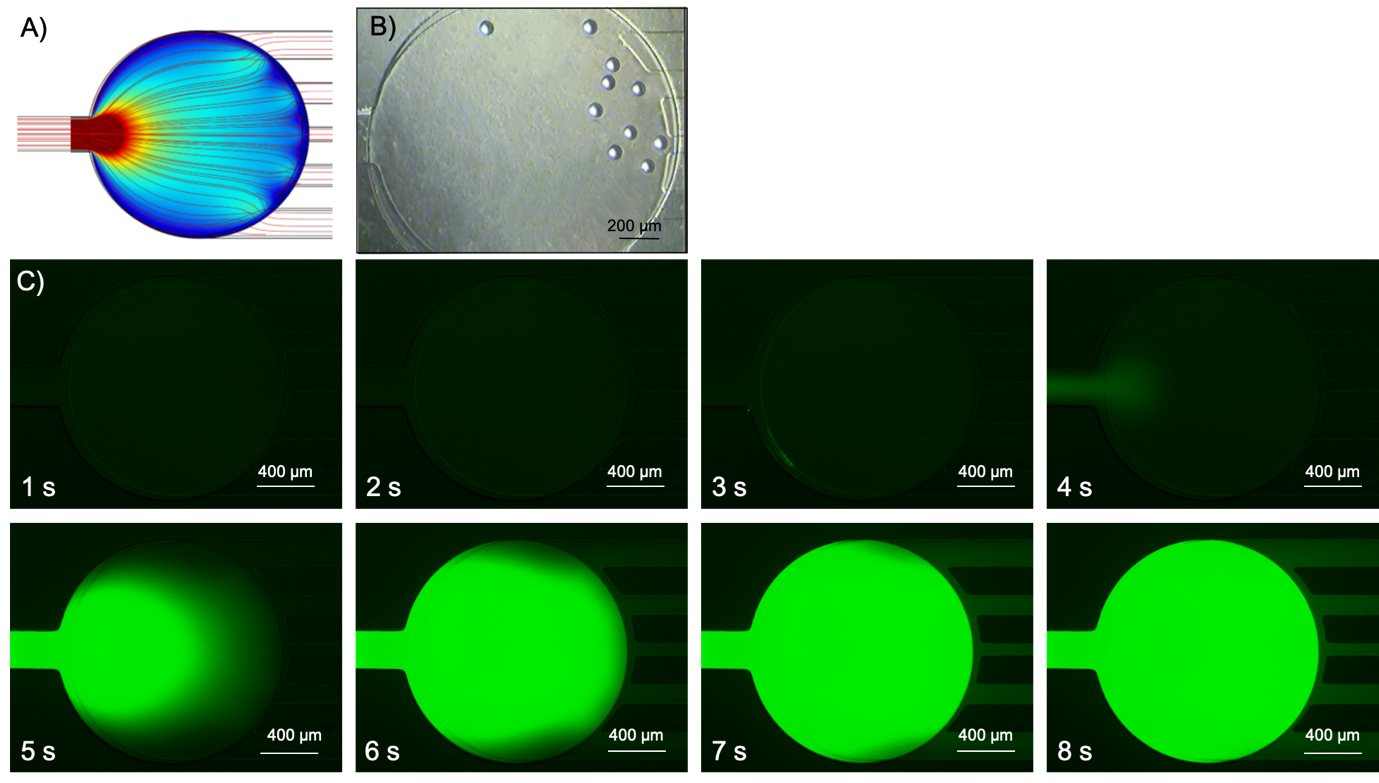
**

**Figure S2**. Real flow characterization within the culture chamber. A) Velocity magnitude surface and stream lines plot. B) Polystyrene beads spread within the culture chamber. C) Microfluidic device filled with a 0.05 mg ml^-1^ fluorescein solution.

**Figure S3**

|  | Control | | | Device | | |
| --- | --- | --- | --- | --- | --- | --- |
| Blastocyst Stage | Mean | SD | N | Mean | SD | N |
| Early Blastocyst | 5.67 | 10.92 | 7 | 6.61 | 6.72 | 19 |
| Blastocyst | 7.68 | 8.01 | 7 | 12.86 | 10.21 | 19 |
| Expanded Blastocyst | 17.39 | 18.67 | 7 | 15.47 | 16.42 | 19 |
| Hatching Blastocyst | 26.72 | 26.74 | 7 | 17.13 | 15.32 | 19 |
| Hatched Blastocyst | 9.18 | 18.77 | 7 | 12.00 | 16.05 | 19 |

**Figure S3**: Representative comparison of blastocyst rate and hatching between embryos developed in the microdrop controls (▲) and in the microfluidic devices (■). The differences are not significant (2-way ANOVA p=0.86), confirming that the there are no difference in timing of development, and the value of the device as a valid alternative to current culture methods

**Figure S4**
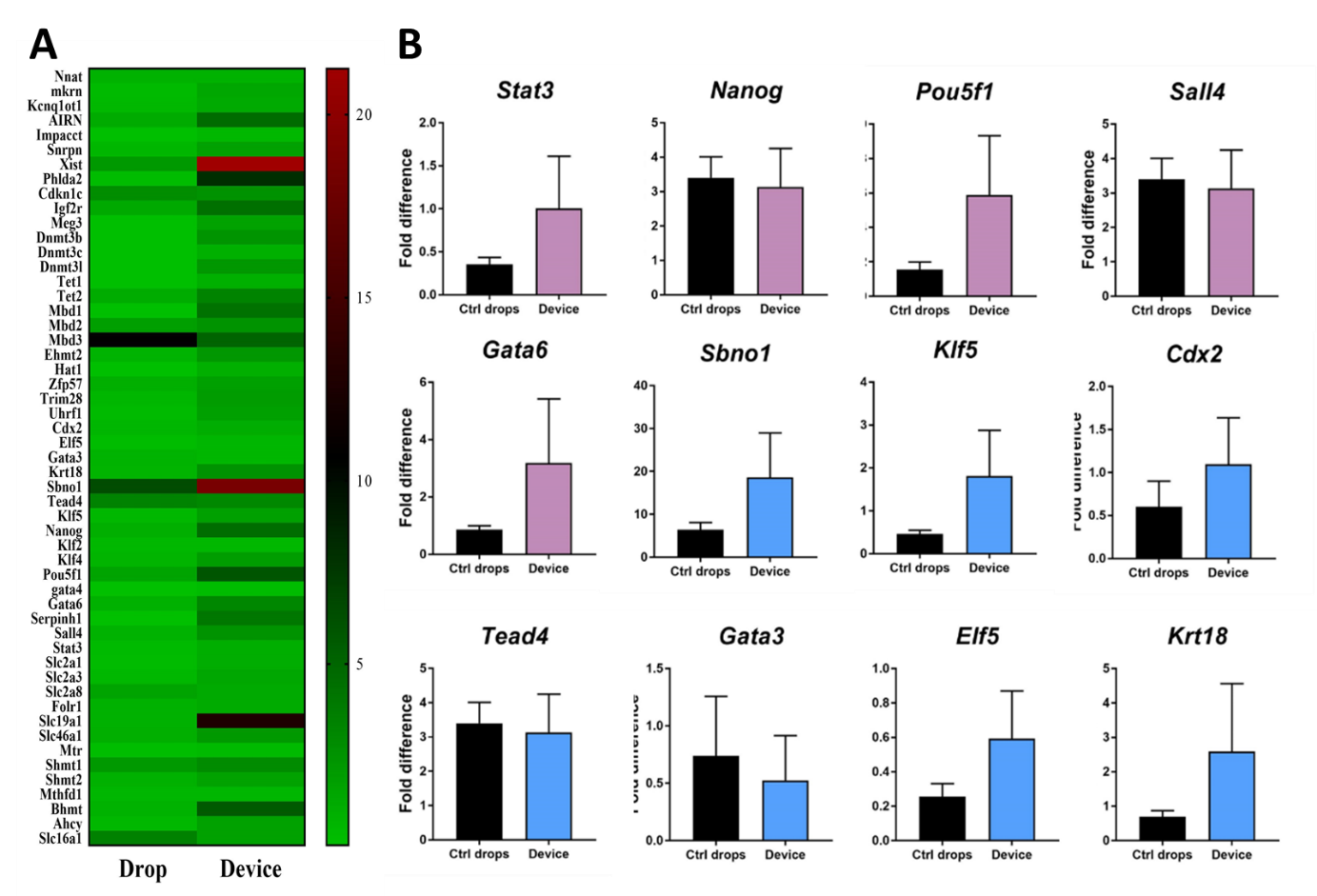


**Figure S4.** Relative mRNA expression of selected genes (purple: markers of ICM/epiblast differentiation; blue: markers of trophectoderm differentiation) in blastocysts (n=10) cultured in the device, compared with control (n=10). Data are presented as mean ± SEM for the numbers of embryos shown.

**Figure S5**


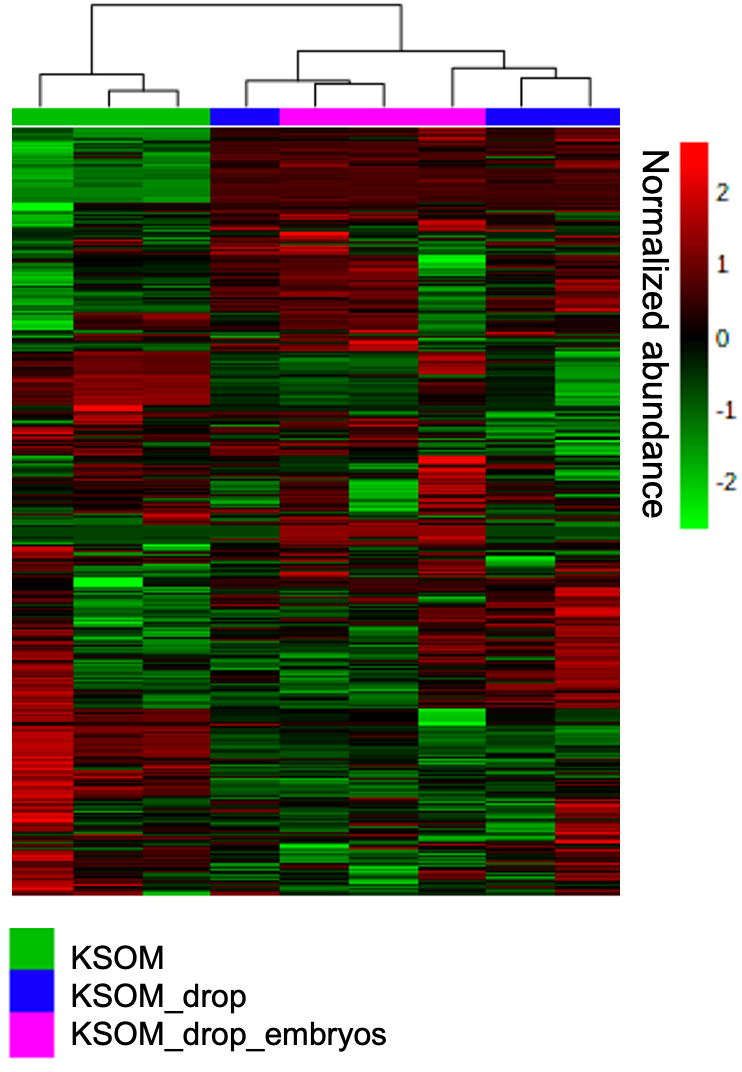


**Figure S5**. Heatmap analysis of media samples collected from control microdrop with and without embryos. Sample replicates are visualized in columns column based on hierarchical clustering, with metabolites presented on individual rows. Species are colored based on normalized abundance from red (high) to green (low).

*Table S1. Summary of gene symbol, accession number, product length and primer sequences of the genes library used in this work.*

| **Symbol** | **Name** | **Accession** | **Size (bp)** | **Forward** | **Reverse** |
| --- | --- | --- | --- | --- | --- |
| Nnat | neuronatin | NM_010923 | 174 | TTGAATCCCACCTTTACCAA | CCTGTCTCCAGGAGCTTACA |
| Mkrn | Makorin, ring finger protein, 1 | NM_018810 | 218 | TTGTGGGTATCTGCCTCATT | AGAGCAACAAAATGCAAAGG |
| Kcnq1ot1 | KCNQ1 overlapping transcript 1 | NR_001461 | 234 | CCCAAACCTTAGTCCTCCAT | TAACAAAGGGCACACGGTAT |
| AIRN | antisense Igf2r RNA | NR_002853 | 111 | AGCTTGATCCACTTGCAAAC | CTGGATGTCCCTGTTAGGTG |
| Impact | impact, RWD domain protein | NM_008378 | 227 | TACAGCACCGCCTTCTTATC | CTTCTTCGGACTCAACCTCA |
| Snrpn | small nuclear ribonucleoprotein N | NM_013670 | 202 | AAAACAGCCAGAACGTGAAG | AGCCTGGGGAATAGGTACAC |
| Xist | In multiple Geneids | X59289 | 161 | TGTGGTCTGCTTTGTCTTCA | ACAAAGAACAAGTGGGGTGA |
| Phlda2 | pleckstrin homology-like domain, family A, member 2 | NM_009434 | 69 | CGTGGAGCACACCTCTAAGT | CGGAAGTCGATCTCCTTGTA |
| Cdkn1c | cyclin-dependent kinase inhibitor 1C (P57) | NM_001161624 | 185 | TCACCAGCTTCAGATTACCC | GTTGGTATGGGCAGTACAGG |
| Igf2r | insulin-like growth factor 2 receptor | NM_010515 | 174 | CTGCATTGGCTTGTACTCCT | TATAAGGCAGAGGGTTGCAG |
| Meg3 | maternally expressed 3 | NR_003633 | 152 | TTAGCAGCAGTGGACATGAA | GGCTGTGAAGGAAAGACAGA |
| Dnmt3b | DNA methyltransferase 3B | NM_001003961 | 155 | ACTTGGTGATTGGTGGAAGC | CCAGAAGAATGGACGGTTGT |
| Dnmt3c | Aka Sycp1 | XM_021192798 | 241 | TGATGATCAATGCCATCAAG | ATGTCGTCTTTGCCATTCAT |
| Dnmt3l | DNA (cytosine-5-)-methyltransferase 3-like | NM_019448 | 179 | CATCCCTACCTACGGGTTCT | AGAGGACTGTCAGCATCAGG |
| Tet1 | tet methylcytosine dioxygenase 1 | NM_001253857 | 142 | AAGAAGAGGAAATGCGAGGT | GGCCATTTACTGGTTTGTTG |
| Tet2 | tet methylcytosine dioxygenase 2 | NM_001040400 | 200 | ATGCAGAGAGATGCCTTCAC | GCCGTGTAGCTGTAGATCGT |
| Mbd1 | methyl-CpG binding domain protein 1 | NM_013594 | 84 | AAAGAGGACTGTGGGGTGTG | CCGCTCACACTTGCAGTAAA |
| Mbd2 | methyl-CpG binding domain protein 2 | NM_010773 | 205 | AGACCCTTCTGTCTGCTGTG | AGTGCCTCCTCCAGTTTCTT |
| Mbd3 | methyl-CpG binding domain protein 3 | NM_013595 | 246 | GTGGATTGAGTGCCTTTGAC | TGATGTCGTCATCTGTCACC |
| Ehmt2 | euchromatic histone lysine N-methyltransferase 2 | NM_147151 | 222 | CCCGTTACTATGGCAACATC | ACTTCTCAGAGCCACACTGG |
| Hat1 | Histone acetyltransferase 1 | NM_026115 | 150 | GTGCCGTGGAGAAGAAACTA | CAGTTTCATCATCCCCAAAG |
| Zfp57 | zinc finger protein 57 | NM_001013745 | 85 | TTAACCCACCTCAAGATCCA | TGAGTGTGTTGGATGAGTGG |
| Trim28 | tripartite motif-containing 28 | NM_011588 | 166 | AGTAAGTGGGCCAGGTATCC | GCAGAGTGAGCAACTCCATT |
| Uhrf1 | ubiquitin-like, containing PHD and RING finger domains, 1 | NM_010931 | 150 | ATGGCCAACTATAACGTGGA | CATGATCCGACAGTTGTTGA |
| Cdx2 | caudal type homeobox 2 | NM_007673 | 150 | TCAGGGGAAGACATGGTTTA | AGGCTGATAGCTTCATGTCG |
| Elf5 | E74-like factor 5 | NM_010125 | 112 | GTGCACCCTGAATACTGGAC | CGCTGATGTTGAAGTGACAG |
| Gata3 | GATA binding protein 3 | NM_008091 | 189 | CCCTTTATTCCTCCGTGTCT | GAGAGGGGTCGTTTAATGGT |
| Krt18 | keratin 18 | NM_010664 | 173 | ACCTGAGGGCTCAGATCTTT | GGTGTCATCTACCACCTTGC |
| Sbno1 | sno, strawberry notch homolog 1 (Drosophila) | NM_001081203 | 193 | GCAGCTGAGCTTTACTGGAG | CTGGTGAGCAGACCAGAACT |
| Tead4 | TEA domain family member 4 | NM_011567 | 62 | TCACCTGCTCTACGAAGGTC | CTCCGTCTCAACTTTCTCCA |
| Klf5 | Kruppel-like factor 5 | NM_009769 | 248 | CACCATGCCAAGTCAGTTTC | TCTCCAGATCCGGGTTACTC |
| Nanog | Nanog homeobox | NM_028016 | 207 | GGACAGGTTTCAGAAGCAGA | CAATGGATGCTGGGATACTC |
| Klf2 | Kruppel-like factor 2 (lung) | NM_008452 | 187 | GGCTAGATGCCTTGTGAGAA | CACCACTACCGTGATTCCTC |
| Klf4 | Kruppel-like factor 4 (gut) | NM_010637 | 232 | TCAAGTTCCCAGCAAGTCAG | AAACTTCCAGTCACCCCTTG |
| Pou5f1 | POU domain, class 5, transcription factor 1 | NM_013633 | 160 | AAGCAACTCAGAGGGAACCT | GGTGATCCTCTTCTGCTTCA |
| Gata4 | GATA binding protein 4 | NM_008092 | 246 | TCTCACTATGGGCACAGCAG | CGAGCAGGAATTTGAAGAGG |
| Gata6 | GATA binding protein 6 | NM_010258 | 168 | TAGAAATGCTGAGGGTGAGC | ACAGAGCCACTGCTGTTACC |
| Serpinh1 | serine (or cysteine) peptidase inhibitor, clade H, member 1 | NM_009825 | 239 | GTGTCACTGGGTGGTAAAGC | TTGGAGTGTTCGCAGTTGTA |
| Sall4 | sal-like 4 (Drosophila) | NM_175303 | 125 | TTAAGCAGCCATGTGTCTCA | GGTAGCTTGGCTTGTTTCAA |
| Stat3 | signal transducer and activator of transcription 3 | NM_213659 | 187 | ATCTGTAACCACAGGGCAAA | GTAAGCTGAGTGAGCGAAGC |
| Slc2a1 | solute carrier family 2 (facilitated glucose transporter), member 1 | NM_011400 | 163 | TGCCAAGCTAATCTGTAGGG | GAATGGGCGAATCCTAAAAT |
| Slc2a3 | solute carrier family 2 (facilitated glucose transporter), member 3 | NM_011401 | 204 | TTTTCCACAGGTCACTGGAT | GTTAGAGGAGTCGCCTTTCC |
| Slc2a8 | solute carrier family 2, (facilitated glucose transporter), member 8 | NM_019488 | 197 | CGCCTGTACCTCACTTGACT | GGAAAGAGCCAGAAAGAACC |
| Folr1 | folate receptor 1 | NM_001252552 | 163 | AAGAGGACTGTCAGCAGTGG | TCACACAGAGCAGCAGATGT |
| Slc19a1 | solute carrier family 19 (folate transporter), member 1 | NM_031196 | 85 | GGAGCAGGTGACTAACGAGA | GTAGTCGGTGAGCAGGAAGA |
| Slc46a1 | solute carrier family 46, member 1 | NM_026740 | 137 | GTTCCAGCAGTTTCCTCAGA | CAAGGGTGGATAGACTGTGG |
| Mtr | 5-methyltetrahydrofolate-homocysteine methyltransferase | NM_001081128 | 144 | GAGGAAGGACAAGCAACAGA | TTCCTCCATTCCAGTACCAA |
| Shmt1 | serine hydroxymethyltransferase 1 | NM_009171 | 125 | TCTTTGGTGGGTGTTTCTGT | AATAGCTTTGCCTCACATGC |
| Shmt2 | serine hydroxymethyltransferase 2 | NM_028230 | 102 | GCTCAAAGACCCAGAGACAA | CCTCAACGTTCATCAAATCC |
| Mthfd1 | Methylenetetrahydrofolate dehydrogenase (NADP+ dependent), methenyltetrahydrofolate cyclohydrolase, formyltetrahydrofolate synthase | NM_138745 | 213 | ATTGCACTCAAGCTGGTAGG | TGTGTAAGCCTTGGGAAGAG |
| Bhmt | Betaine-homocysteine methyltransferase | NM_016668 | 157 | TGTGGCAAACAGGGATTTAT | TGATGTGGTAGGGCTCAAAT |
| Ahcy | S-adenosylhomocysteine hydrolase | NM_016661 | 193 | AGCTGAATGTGAAGCTGACC | AGGCTACTGCAAAGTTGGTG |
| Slc16a1 | solute carrier family 16 (monocarboxylic acid transporters), member 1 | NM_009196 | 156 | AGAGGTTCTCCAGTGCTGTG | GATAGATACCCGCGATGATG |

*Table S2. List of compounds that were significantly (p<0.05, FC>2) up-regulated uniquely in day 5 embryo culture PDMS-media vs day 0 KSOM.*

| (-)-11-hydroxy-9,10-dihydrojasmonic acid 11-beta-D-glucoside  (-)-AS 115 22453  (-)-trans-Carveol glucoside  (+)-Mahanimbicine  (+)-Neoisomenthol  (1S,2R,4R,8S)-p-Menthane-2,8,9-triol 2-glucoside  (2E)-hexenedioylcarnitine  (2R,3R)-heptane-1,2,3-triol  (3R,7R)-1,3,7-Octanetriol  (3S,4S)-3-hydroxytetradecane-1,3,4-tricarboxylic acid  (4-ethyl-2-methoxyphenyl)oxidanesulfonic acid  (4-Methylphenyl)acetaldehyde  (4E)-6-hydroxy-1-(4-hydroxy-3-methoxyphenyl)tetradec-4-en-3-one  (5-Chloro-1H-indol-2-yl)(4-methyl-1-piperazinyl)methanone  (S)-2-Acetolactate  (S)-3-Mercaptohexyl pentanoate  (S)-a-Amino-2,5-dihydro-5-oxo-4-isoxazolepropanoic acid N2-glucoside  (S)-Homostachydrine  (S)-Reticuline  (S1)-Methoxy-3-heptanethiol  {[(1E)-5-oxo-1,7-diphenylhept-1-en-3-yl]oxy}sulfonic acid  {2-hydroxy-5-[3-(4-hydroxyphenyl)propanoyl]phenyl}oxidanesulfonic acid  1-(sn-Glycero-3-phospho)-1D-myo-inositol  1-[2-Methyl-3-(methylthio) allyl]cyclohex-2-enol  1-Ipomeanol  1-Isothiocyanato-2-phenylethane  1,2-Dehydrosalsolinol  1,3-Dimethyluric acid  1,7-Dimethylguanosine  10-Hydroxy-8-nor-2-fenchanone glucoside  17-phenyl trinor Prostaglandin E2 serinol amide 20818  2-(1-Ethoxyethoxy)propanoic acid  2-{2-[(6-carboxy-3,4,5-trihydroxyoxan-2-yl)oxy]phenyl}-1Î»â´-chromen-1-ylium  2-Acetyl-3,5-dimethylpyrazine  2-amino-4-({1-[(carboxymethyl)-C-hydroxycarbonimidoyl]-2-[(1,3-dihydroxy-1-phenylpropan-2-yl)sulfanyl]ethyl}-C-hydroxycarbonimidoyl)butanoic acid  2-amino-4-({1-[(carboxymethyl)-C-hydroxycarbonimidoyl]-2-[(1,3-dihydroxy-2-methyl-1-phenylpropan-2-yl)sulfanyl]ethyl}-C-hydroxycarbonimidoyl)butanoic acid  2-amino-4-({1-[(carboxymethyl)-C-hydroxycarbonimidoyl]-2-{[2-hydroxy-3-(4-hydroxy-2-methoxyphenyl)-1-phenylpropyl]sulfanyl}ethyl}-C-hydroxycarbonimidoyl)butanoic acid  2-amino-4-({2-[(2-carboxy-2-hydroxy-1-phenylethyl)sulfanyl]-1-[(carboxymethyl)-C-hydroxycarbonimidoyl]ethyl}-C-hydroxycarbonimidoyl)butanoic acid  2-Amino-5-benzoylbenzimidazole  2-Aminoheptanoate  2-hydroxy-7-methyl-Octanedioic acid  2-Hydroxyfelbamate  2-O-Methyl-D-xylose  2-Phthalimidoglutaric acid  2-Piperidinone  2,3-Butanediol glucoside  3-Cyano-4,7-dimethylcoumarin  3-Deoxy-D-glycero-D-galacto-2-nonulosonic acid  3-HYDROXY-3-METHYLGLUTARATE-16  3-hydroxyhexanoyl carnitine  3-Hydroxymethylglutaric acid  3-hydroxynonanoyl carnitine  3-Isopropenylpentanedioic acid  3-Mercapto-3-methylbutyl formate  3-Methyl-.gamma.-butyrolactone  3-Methylxanthine  3-O-Methyl-a-methyldopa  3-octenoylglycine  3-Oxo-1,8-octanedicarboxylic acid  3,4-Methyleneadipic acid  3,5-Bis(trifluoromethyl)diphenylamine  3,7,8,15-Scirpenetetrol  3''-Chloro-3''-deoxytriphasiol  4-{1-hydroxy-3-[4-hydroxy-2-methoxy-3-(3-methylbut-2-en-1-yl)phenyl]propyl}benzene-1,2-diol  4-Butyl-5-ethylthiazole  4-Heptenoic acid  4-hydroxy-2-methylbut-2-enoic acid  4-hydroxyoct-5-enoylglycine  4-Methyldibenzothiophene  4-O-Methylmelleolide  4-octenoylglycine  4-Phosphopantothenoylcysteine  5-Geranyloxy-7-methoxycoumarin  5-Hexyltetrahydro-2-oxo-3-furancarboxylic acid  5-Hydroxy-6-methoxycoumarin 7-glucoside  5-Hydroxy-7-(4-hydroxy-3-methoxyphenyl)-1-phenyl-3-heptanone  5-Hydroxy-L-tryptophan  5-Methoxytryptophan  5-Nitrobarbituric acid  5-octenoylglycine  6-(1-hydroxy-2-methylbut-3-en-2-yl)-2-(2-hydroxypropan-2-yl)-2H,3H,7H-furo[3,2-g]chromen-7-one  6-(Hydroxymethyl)-2,4(1H,3H)-pteridinedione  6-{[3-(6,7-dimethoxy-2H-1,3-benzodioxol-5-yl)prop-2-enoyl]oxy}-3,4,5-trihydroxyoxane-2-carboxylic acid  6-Hydroxyfluvastatin  6Z-8-Hydroxygeraniol 8-O-glucoside  7-Aminoflunitrazepam  7-Aminonitrazepam  7-Hydroxyoctanoic acid  7-Isothiocyanato-1-heptene  7-octenoylglycine  8-Deoxy-11,13-dihydroxygrosheimin  8-Hydroxy-5,6-octadienoic acid  8-iso Prostaglandin F1a-d9 28742  9-(beta-D-Ribofuranosyl)zeatin  9-Methylxanthine  9,9-dimethoxy-nonanoic acid  Abiraterone sulfate  Abu-Lys-OH 773  Acetaldehyde  Acetazolamide  Acetylglycine  Acuminoside  Ajmaline  Ala Asn Met 11107  Ala Ile Ser 11844  Ala Lys Phe 11848  Alfuzosin  Alizapride  Allyl thiohexanoate  Amiprilose 31522  Arginyl-Arginine  Arginyl-Hydroxyproline  Arginyl-Isoleucine  Arginyl-Phenylalanine  Arginyl-Valine  Armillaripin  Asn-Arg-Arg  Asn-Ser-Arg  Asp Asn Val 1875  Asp His Met 12861  Asp Trp Met 13629  Asparaginyl-Valine  Asymmetric dimethylarginine  Benzylamine  Beta-Guanidinopropionic acid  Bis (2-hydroxypropyl) amine 26447  Brassicanal B  Bumetanide  Butyl ethyl malonate  Butyl lactate  Butyramide  Caffeoyl tyrosine  Calystegine B5  Capryloylglycine  Carnitine-d3  Casomorphin  Chitotriose  CHOLINE-41  Chrysophanol 1-triglucoside  Cibulins  Ciclopirox 21996  cis-4-Hydroxyproline  Clausarinol  Cys Glu Phe 13468  Cys Phe Ser 4336  Cysteinyl-Gamma-glutamate  D-Glucosaminic acid  D-Glucosaminide  D-glycero-L-galacto-Octulose  Dehydromatricaric acid  Dendryphiellic acid B  Deoxyribose  Deoxythymidine diphosphate-l-rhamnose  Dexfenfluramine  Diacetone-D-galacturonic acid  Dibromodichloromethane  Dibutyl malate  Didesmethyl doxepin  Diethyl oxalpropionate  Dihydro-2,4,6-tris(2-methylpropyl)-4h-1,3,5-dithiazine  Dihydroergocornine  Dihydrolipoamide  Dimethicone  Dimethyl sulfoxide  Divinyl sulfide  dUDP  Echitamine  Echothiophate  Erinapyrone A  Etamiphylline  Ethanol, 2-[2-(2-butoxyethoxy)ethoxy]-  Ethiofencarb  Fluconazole  Fluoxymesterone  Flupirtine  Furcelleran  Gamma-Butyrolactone  Gamma-glutamyl-L-putrescine  gamma-Glutamyl-S-methylcysteine sulfoxide  gamma-Glutamylisoleucine  gamma-Glutamylleucine | gamma-Glutamylvaline  Gemcitabine  Gingerenone B  Gln Cys Tyr 4996  Gln Ile Lys 7055  Gln-Cys-Lys  Gln-Ser-Arg  Glu Ile Asn 9712  Glu Met Tyr 4441  Glutamylglutamine  Glutamylmethionine  Glutamylvaline  Glutarylcarnitine  Glutarylglycine  Gly Leu 19822  Gly Phe Val 10506  Glycerol  Glycerol 1-propanoate  Glycerol tributanoate  Glycerol tripropanoate  Glycyltyrosine  Hexaethylene glycol  Hexanethioic acid S-propyl ester  His Gln Ala 4582  His Ile Phe 3204  His Ile Val 9187  His Val Val 3965  Histidinyl-Lysine  Homocysteinesulfinic acid  HoPhe-Nap-OH 1154  Hordatine B  Hydromorphone-3-glucuronide  Hydroxyhexanoycarnitine  hydroxyisovaleroyl carnitine  Hydroxyprolyl-Isoleucine  Hydroxypropionylcarnitine  Ile Pro Ala 13948  Indane  Indoleacetaldehyde  Isoleucyl-Isoleucine  Isoleucylproline  Isopropyl beta-D-glucoside  Isovalerylalanine  Itaconic acid  Kyotorphin  L-(-)-Arabitol, permethyl-  L-Acetylcarnitine  L-Aspartate-semialdehyde  L-cis-Cyclo(aspartylphenylalanyl)  L-Cystathionine  L-Formylkynurenine  L-Glutamine  L-glycyl-L-hydroxyproline  L-Hexanoylcarnitine  L-Kynurenine  L-Lysopine  L-Methionine  L-Tryptophan  Lepidine D  Levonordefrin  Lipoyllysine  LMFA01060168  LMFA05000153  LMFA07040007  LMFA08030004  LMFA13010039  LMPK12140461  Lys Tyr Ala 14039  Lys-Ser-Lys  Lys-Val-Lys  Lysyl-Histidine  Margaric acid(d3)  Meglumine  Met(O)-Lys-Arg  Metanephrine  Methionyl-Methionine  Methionyl-Phenylalanine  Methyl Arachidonyl Fluorophosphonate 19691  Meticillin  Mitomycin  Mytilin B  N-.alpha.-(tert-Butoxycarbonyl)-L-Histidine  N-(1-Deoxy-1-fructosyl)glycine  N-(1-Deoxy-1-fructosyl)isoleucine  N-(1-Deoxy-1-fructosyl)leucine  N-(1-Deoxy-1-fructosyl)phenylalanine  N-(1-Deoxy-1-fructosyl)tyrosine  N-(1-Deoxy-1-fructosyl)valine  N-(2-phenoxy-ethyl) arachidonoyl amine  N-(3-aminopropyl)-3-(3,4-dihydroxyphenyl)propanimidic acid  N-(3-Oxooctanoyl)-L-homoserine lactone  N-[2-(3,4-dimethoxyphenyl)ethyl]-3-(3-hydroxy-4-methoxyphenyl)propanimidic acid  N-Acetyl desmethyl frovatriptan  N-Acetyl-L-alanine  N-Acetylarylamine  N-Acetylisoleucine  N-Acetylornithine  N-acetyltryptophan  N-Alpha-acetyllysine  N-Decanoylglycine  N-Fluorenylacetamide  N-Heptanoylglycine  N-m-Tolyloxyacetyl-benzenesulfonamide  N-Methoxyspirobrassinol  N-Methyl-1-deoxynojirimycin  N-Methylethanolaminium phosphate  N-Nonanoylglycine  N-Phenylacetylglutamic acid  N,N-Bis(2-hydroxyethyl)-2-aminoethanesulfonic acid  N,N-Dimethylformamide  N1,N8-Diacetylspermidine  N2-Maltulosylarginine  N5-Carboxyaminoimidazole ribonucleotide  N8-Acetylspermidine  Nap-Thr-OH 626  Necatorine  Neosaxitoxin  Nepafenac  Netilmicin  Nigakinone  Norfenfluramine  O-Acetylserine  O-sebacoylcarnitine  Orciprenaline  Pantothenamide  Pantothenic acid  Pantothenol  Penciclovir  Penicillin G  Penicillin V  Pentaethylene glycol  Pentapropylene glycol  Phaseolic acid  Phe Gly Val 10456  Phe Tyr Val 20210  Phenylalanyl-Arginine  Picrocrocin  Pirbuterol  Piromidic Acid 17891  Portuloside A  Prenyl glucoside  Pro Lys Asn 10821  Pro-Pro-Lys  Propafenone  Propionylcarnitine  Pseudoecgonine  Pterolactam  Pyridine N-oxide glucuronide  Pyridoxamine  PyroGlu-Lys-Lys  Quinaprilat  Quinone  R-2-Hydroxy-3-methylbutanoic acid 3-Methylbutanoyl  S-(3-Methyl-2-butenyl) 2-methylpropanethioate  S-Adenosylmethionine  S-Propyl 1-propanesulfinothioate  SAICAR  Ser Asn Asn 16756  Seryllysine  Spermidine  Sphingosine 1-phosphate (d19:1-P)  Succinic acid  Succinylacetone  Sufentanil  Sulfadiazine  Sulfallate  Swainsonine  Tetraethylene glycol  Tetrapropylene glycol  Theobromine  Thiiranebutanenitrile  Thorium  Thymine  Toxin T2 tetrol  trans-S-(1-Propenyl)-L-cysteine  Triethyl citrate  Triethylene glycol  Trifluridine  Tripropylene glycol  Tyrosine methylester  Tyrosyl-Hydroxyproline  Tyrosyl-Tyrosine  Val Pro His 10192  Val Pro Leu 2286  Val-Gly-Val  Valyl-Valine  Xanthine  xi-2,5-Dihydro-2,4-dimethylthiazole  xi-4-Hydroxy-4-methyl-2-cyclohexen-1-one  Zanamivir  Zapotidine |
| --- | --- |

*Table S3. List of compounds that were significantly (p<0.05, FC>2) up-regulated uniquely in day 4 embryo culture PS-media vs day 0 KSOM.*

| (9xi,10xi,12xi)-9,10-Dihydroxy-12-octadecenoic acid  (Z)-1-(Methylthio)-5-phenyl-1-penten-3-yne  {[3,4,5-trihydroxy-5-(hydroxymethyl)oxolan-2-yl]methoxy}sulfonic acid  1-(4-Fluorobenzyl) piperazine 28321  1-(4-hydroxyphenyl)-7-phenylheptane-3,5-dione  1,2,3-Tris(1-ethoxyethoxy)propane  1,2,3,4-Tetrahydro-b-carboline-1,3-dicarboxylic acid  1,9-Nonanedithiol  15(R)-hydroperoxy-EPE  18-Carboxy-dinor-LTE4  2-Hydroxyglutarate  2-Methoxy-3-methyl-9H-carbazole  2,4-Dimethylpyridine  3-[3,4-dihydroxy-2-(5-hydroxy-3,7-dimethylocta-2,6-dien-1-yl)phenyl]propanoic acid  3-Carbamoyl-2-phenylpropionic acid  3-Deaza-2'-deoxyadenosine 28523  3,4,5-trihydroxy-6-[(2-methylpropanoyl)oxy]oxane-2-carboxylic acid  3,4,5-trihydroxy-6-{[3,6,7-trihydroxy-2-(4-hydroxyphenyl)-4-oxo-3,4-dihydro-2H-1-benzopyran-5-yl]oxy}oxane-2-carboxylic acid  3b,8b-Dihydroxy-6b-(3-chloro-2-hydroxy-2-methylbutanoyloxy)-7(11)-eremophilen-12,8-olide  4-Ethyl-2-methylthiazole  4-Methylbenzoic acid  4,8 Dimethylnonanoyl carnitine  5-Hydroxyomeprazole  5-Oxoprolinate  Abu-Phe-OH 156  Acetylpterosin C  Ala Lys Gln 2386  Alanyl-Arginine  Alpha-Lactose  Arginyl-Asparagine  Arginyl-Glycine  Arginyl-Lysine  Arginyl-Tyrosine  Aspartylphenylalanine  Avenanthramide C  Benzeneacetamide-4-O-sulphate | Carboxytolbutamide  Chrycorin  Cilastatin  cis-2-Methylaconitate  Dimethylallylpyrophosphate  Eriobofuran  Flavoxate  Gancaonin A  Histidinyl-Hydroxyproline  Homolanthionine  Hydroxyprolyl-Threonine  Hydroxytyrosol 1-O-glucoside  Indanone  Isogemichalcone B  Isoginkgetin  KH7 23352  L-2-Amino-3-(oxalylamino)propanoic acid  L-Proline  Lanthionine ketimine  Leucyl-Leucine  Maltol  Meropenem  Met Thr Tyr 14215  Mevalonic acid  N-(4-aminobutyl)-3-(4-hydroxy-3-methoxyphenyl)oxirane-2-carboximidic acid  N-Caffeoyltryptophan  Nb-Feruloyltryptamine  O-SUCCINYL-L-HOMOSERINE-34  Ornithine  Parvisoflavone A  Pinocembrin 7-apiosyl-(1->5)-apiosyl-(1->2)-glucoside  S-Allylcysteine  Saccharopine  Talaromycin A  Theaflavin  Tryptophyl-Gamma-glutamate  Tyramine glucuronide  Valyl-Isoleucine |
| --- | --- |

*List of compounds that were significantly (p<0.05, FC>2) down-regulated uniquely in day 5 embryo culture PDMS-media vs day 0 KSOM.*

| Carboxytolbutamide  Chrycorin  Cilastatin  cis-2-Methylaconitate  Dimethylallylpyrophosphate  Eriobofuran  Flavoxate  Gancaonin A  Histidinyl-Hydroxyproline  Homolanthionine  Hydroxyprolyl-Threonine  Hydroxytyrosol 1-O-glucoside  Indanone  Isogemichalcone B  Isoginkgetin  KH7 23352  L-2-Amino-3-(oxalylamino)propanoic acid  L-Proline  Lanthionine ketimine  Leucyl-Leucine | Maltol  Meropenem  Met Thr Tyr 14215  Mevalonic acid  N-(4-aminobutyl)-3-(4-hydroxy-3-methoxyphenyl)oxirane-2-carboximidic acid  N-Caffeoyltryptophan  Nb-Feruloyltryptamine  O-SUCCINYL-L-HOMOSERINE-34  Ornithine  Parvisoflavone A  Pinocembrin 7-apiosyl-(1->5)-apiosyl-(1->2)-glucoside  S-Allylcysteine  Saccharopine  Talaromycin A  Theaflavin  Tryptophyl-Gamma-glutamate  Tyramine glucuronide  Valyl-Isoleucine |
| --- | --- |

*List of compounds that were significantly (p<0.05, FC>2) down-regulated uniquely in day 4 embryo culture PS-media vs day 0 KSOM.*

| Carboxytolbutamide  Chrycorin  Cilastatin  cis-2-Methylaconitate  Dimethylallylpyrophosphate  Eriobofuran  Flavoxate  Gancaonin A  Histidinyl-Hydroxyproline  Homolanthionine  Hydroxyprolyl-Threonine  Hydroxytyrosol 1-O-glucoside  Indanone  Isogemichalcone B  Isoginkgetin  KH7 23352  L-2-Amino-3-(oxalylamino)propanoic acid  L-Proline  Lanthionine ketimine  Leucyl-Leucine | Maltol  Meropenem  Met Thr Tyr 14215  Mevalonic acid  N-(4-aminobutyl)-3-(4-hydroxy-3-methoxyphenyl)oxirane-2-carboximidic acid  N-Caffeoyltryptophan  Nb-Feruloyltryptamine  O-SUCCINYL-L-HOMOSERINE-34  Ornithine  Parvisoflavone A  Pinocembrin 7-apiosyl-(1->5)-apiosyl-(1->2)-glucoside  S-Allylcysteine  Saccharopine  Talaromycin A  Theaflavin  Tryptophyl-Gamma-glutamate  Tyramine glucuronide  Valyl-Isoleucine |
| --- | --- |

**Video 1** Loading <https://youtu.be/hGSYNKFJsqM>

**Video 2** Retrieval <https://youtu.be/q--bm8kyIJs>
